# Revealing evolutionary constraints on proteins through sequence analysis

**DOI:** 10.1371/journal.pcbi.1007010

**Published:** 2019-04-24

**Authors:** Shou-Wen Wang, Anne-Florence Bitbol, Ned S. Wingreen

**Affiliations:** 1 Department of Engineering Physics, Tsinghua University, Beijing, China; 2 Beijing Computational Science Research Center, Beijing, China; 3 Lewis-Sigler Institute for Integrative Genomics, Princeton University, Princeton, New Jersey, United States of America; 4 Sorbonne Université, CNRS, Laboratoire Jean Perrin (UMR 8237), F-75005 Paris, France; 5 Department of Molecular Biology, Princeton University, Princeton, New Jersey, United States of America; University of Texas at Dallas, UNITED STATES

## Abstract

Statistical analysis of alignments of large numbers of protein sequences has revealed “sectors” of collectively coevolving amino acids in several protein families. Here, we show that selection acting on any functional property of a protein, represented by an additive trait, can give rise to such a sector. As an illustration of a selected trait, we consider the elastic energy of an important conformational change within an elastic network model, and we show that selection acting on this energy leads to correlations among residues. For this concrete example and more generally, we demonstrate that the main signature of functional sectors lies in the small-eigenvalue modes of the covariance matrix of the selected sequences. However, secondary signatures of these functional sectors also exist in the extensively-studied large-eigenvalue modes. Our simple, general model leads us to propose a principled method to identify functional sectors, along with the magnitudes of mutational effects, from sequence data. We further demonstrate the robustness of these functional sectors to various forms of selection, and the robustness of our approach to the identification of multiple selected traits.

## Author summary

Proteins play crucial parts in all cellular processes, and their functions are encoded in their amino-acid sequences. Recently, statistical analyses of protein sequence alignments have demonstrated the existence of “sectors” of collectively correlated amino acids. What is the origin of these sectors? Here, we propose a simple underlying origin of protein sectors: they can arise from selection acting on any collective protein property. We find that the main signature of these functional sectors lies in the low-eigenvalue modes of the covariance matrix of the selected sequences. A better understanding of protein sectors will make it possible to discern collective protein properties directly from sequences, as well as to design new functional sequences, with far-reaching applications in synthetic biology.

## Introduction

Proteins play crucial roles in all cellular processes, acting as enzymes, motors, receptors, regulators, and more. The function of a protein is encoded in its amino-acid sequence. In evolution, random mutations affect the sequence, while natural selection acts at the level of function, however our ability to predict a protein’s function directly from its sequence has been very limited. Recently, the explosion of available sequences has inspired new data-driven approaches to uncover the principles of protein operation. At the root of these new approaches is the observation that amino-acid residues which possess related functional roles often evolve in a correlated way. In particular, analyses of large alignments of protein sequences have identified “sectors” of collectively correlated amino acids [1–6], which has enabled successful design of new functional sequences [3]. Sectors are spatially contiguous in the protein structure, and in the case of multiple sectors, each one may be associated with a distinct role [4, 7]. What is the origin of these sectors, and can we identify them from sequence data in a principled way?

To address these questions, we developed a general physical model that naturally gives rise to sectors. Specifically, motivated by the observation that many protein properties reflect additive contributions from individual amino acids [8–10], we consider any additive trait subject to natural selection. As a concrete example, we study a simple elastic-network model that quantifies the energetic cost of protein deformations [11], which we show to be an additive trait. We then demonstrate that selection acting on any such additive trait automatically yields collective correlation modes in sequence data. We show that the main signature of the selection process lies in the small-eigenvalue modes of the covariance matrix of the selected sequences, but we find that some signatures also exist in the widely-studied large-eigenvalue modes. Finally, we demonstrate a principled method to identify sectors and to quantify mutational effects from sequence data alone.

## Model and methods

### Selection on an additive trait

We focus on selection on an additive scalar trait

$$T\left(\vec{\alpha }\right)=\sum_{l=1}^{L}\Delta_{l}\left(\alpha_{l}\right)\;,$$ (1)

where $\vec{\alpha }=\left(\alpha_{1},\ldots ,\alpha_{L}\right)$ is the amino-acid sequence considered, *L* is its length, and Δ_*l*_(*α*_*l*_) is the mutational effect on the trait *T* of a mutation to amino acid *α*_*l*_ at site *l*. Mutational effects can be measured with respect to a reference sequence ${\vec{\alpha }}^{0}$, satisfying $\Delta_{l}\left(\alpha_{l}^{0}\right)=0$ for all *l*.

Eq 1 is very general as it amounts to saying that, to lowest order, mutations have an additive effect on the trait *T*, which can be any relevant physical property of the protein, say its binding affinity, catalytic activity, or thermal stability [12]. System-specific details are encoded by the single-site mutational effects Δ_*l*_(*α*_*l*_), which can be measured experimentally. The assumption of additivity is experimentally validated in many cases. For instance, protein thermal stability, measured through folding free energy, is approximately additive [8, 13]. Importantly, we allow selection to act on a phenotype that is a nonlinear function of *T*. Permitting a phenotypic nonlinearity on top of our additive trait model is motivated by the fact that actual phenotype data from recent high-throughput mutagenesis experiments were accurately modeled via a nonlinear mapping of an underlying additive trait [10].

Protein sectors are usually defined operationally as collective modes of correlations in amino-acid sequences. However, the general sequence-function relation in Eq 1 suggests an operational definition of a *functional* protein sector, namely as the set of sites with dominant mutational effects on a trait under selection. Selection can take multiple forms. To be concrete, we first consider a simple model of selection, assuming a favored value T* of the trait *T*, and using a Gaussian selection window. We subsequently show that the conclusions obtained within this simple model are robust to different forms of selection. Our Gaussian selection model amounts to selecting sequences according to the following Boltzmann distribution:

$$P\left(\vec{\alpha }\right)=\frac{\text{exp}(w\left(\vec{\alpha }\right))}{\sum_{\vec{\alpha }}\text{exp}\left(w\left(\vec{\alpha }\right)\right)}\;,$$ (2)

where the fitness $w(\vec{\alpha })$ of a sequence is given by

$$w\left(\vec{\alpha }\right)=-\frac{\kappa }{2}{\left(T\left(\vec{\alpha }\right)-T^{*}\right)}^{2}=-\frac{\kappa }{2}(\sum_{l=1}^{L}\Delta_{l}\left(\alpha_{l}\right)-T^{*}{)}^{2}\;.$$ (3)

The selection strength *κ* sets the width of the selection window.

Such selection for intermediate values of a trait can be realistic, e.g. for protein stability [8]. However, the form of selection can vary, for example selection can be for a nonlinear transform of a trait to be above a certain threshold [10], and several relevant selection variants are investigated below. Crucially, while the trait is additive (Eq 1), the fact that fitness (Eq 3) and selection (Eq 2) are nonlinear functions of the trait leads to coupling between mutations. This phenomenon is known as global [10, 14] or nonspecific [9] epistasis, and its relevance has been shown in evolution experiments [14], over and above contributions from specific epistasis [9, 15]. The focus of this paper is on global epistasis, and we do not include specific epistasis. Studying the interplay of these two types of epistasis will be an interesting future direction.

### A toy model yielding a concrete example of an additive trait

#### Elastic-network model

**Fig 1:**
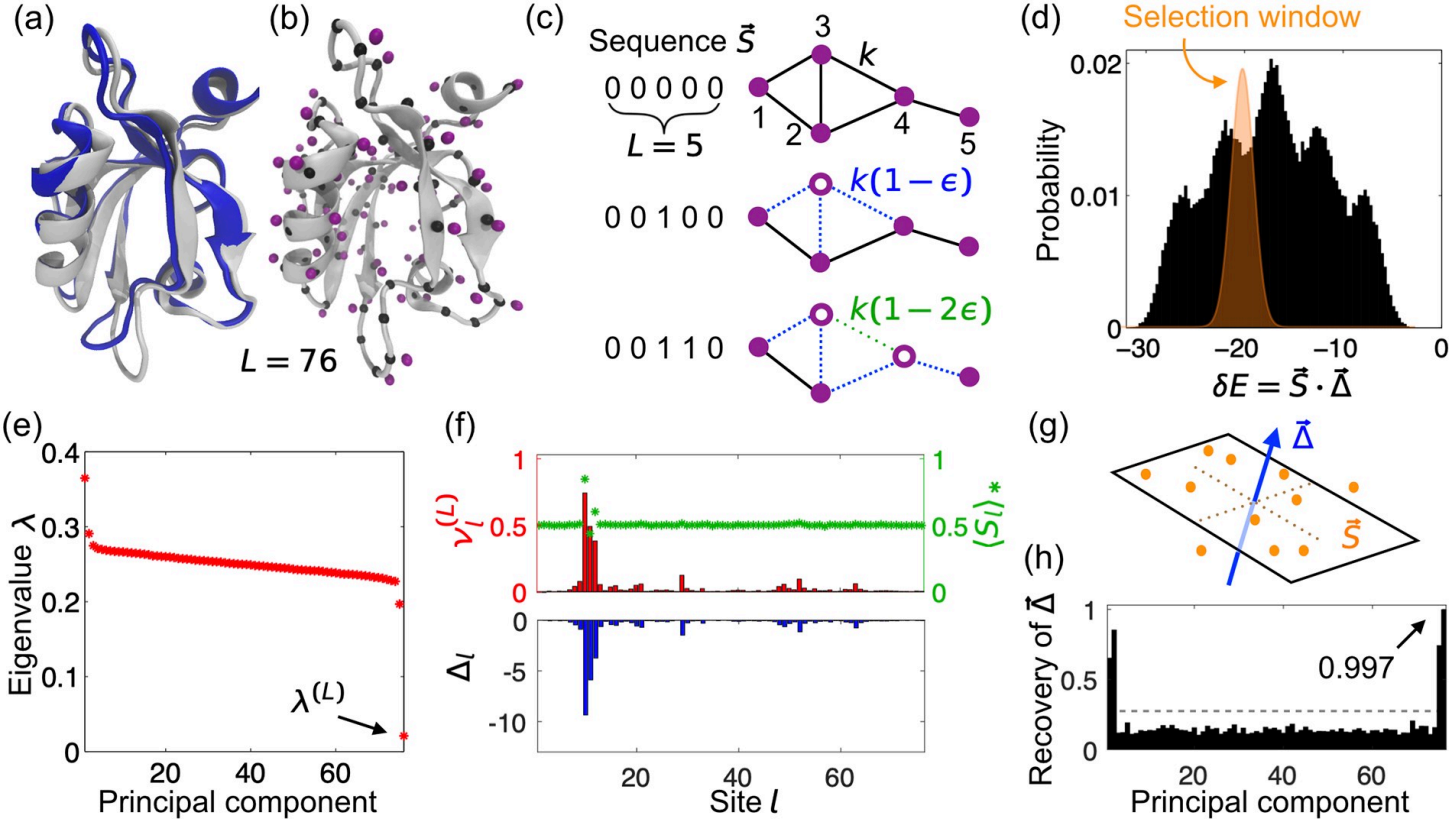
Selection applied to an elastic protein model leads to a statistical signature among sequences. (a) Cartoon representation of the third PDZ domain of the rat postsynaptic density protein 95 from the RSCB PDB [32] (gray: ligand free, 1BFE; blue: ligand bound, 1BE9 (ligand not shown)). (b) Elastic network model for 1BFE, where each amino-acid residue is represented by its alpha carbon (C*α*, black node) and beta carbon (C*β*, purple node). Nearby nodes interact through a harmonic spring [20] (S1 Appendix). (c) Relation between protein sequence $\vec{S}$ and elastic network: 0 denotes the reference state, while 1 denotes a mutated residue, which weakens interactions of the corresponding C*β* with all its neighbors by *ϵ*. (d) Histogram of the energy *δE* required to deform the domain from its ligand-free to its ligand-bound conformation, for randomly sampled sequences where 0 and 1 are equally likely at each site. Sequences are selectively weighted using a narrow Gaussian window (orange) around δE*. (e) Eigenvalues of the covariance matrix *C* for the selectively weighted protein sequences. (f) Upper panel: last principal component $\nu_{l}^{\left(L\right)}$ of *C* (red) and average mutant fraction 〈*S*_*l*_〉_*_ (green) at site *l* after selection; lower panel: effect Δ_*l*_ of a single mutation at site *l* on *δE*. (g) Schematic representation of the selected ensemble in sequence space, where each dot is a highly-weighted sequence; thus dots are restricted to a narrow region around a plane perpendicular to $\vec{\Delta }$. (h) Recovery of $\vec{\Delta }$ for all principal components ${\vec{\nu }}^{\left(j\right)}$, with maximum Recovery = 1 (Eq 8). Gray dashed line: random expectation of Recovery (S1 Appendix).

To illustrate how additive traits naturally arise, we consider the elastic energy associated with a functionally important protein deformation. We explicitly derive the additivity of this trait in the regime of small deformations and weak mutational effects. This concrete example is relevant since functional deformation modes are under selection in proteins [16–18], and dynamical domains possess a signature in sequence data [19]. Moreover, elastic-network models have elucidated a variety of protein properties [11, 20–22], including the emergence of allostery [23–29]. Thus motivated, we begin by building an elastic-network model [11, 20] for a well-studied PDZ protein domain (Fig 1(a) and 1(b)) [30, 31] and computing the relationship between its “sequence” and the energetic cost of a functionally-relevant conformational change.

To build the elastic-network model of the PDZ domain, we replace each of the *L* = 76 amino-acid residues by its corresponding alpha carbon C*α* and beta carbon C*β*, as shown in Fig 1(b). Every pair of carbons within a cutoff distance *d*_*c*_ is then connected with a harmonic spring [11]. Following a previous analysis of the same PDZ domain [20], we set *d*_*c*_ = 7.5 Å and assign spring constants as follows: a) 2 for C*α*-C*α* pairs if adjacent along the backbone, 1 otherwise; b) 1 for C*α*-C*β* pairs; c) 0.5 for C*β*-C*β* pairs.

Within our elastic model, the energetic cost of a small deformation from the equilibrium structure is

$$E=\frac{1}{2}\sum_{i,j}(r_{i}-r_{i}^{0})M_{ij}(r_{j}-r_{j}^{0})=\frac{1}{2}\;\delta r^{T}M\delta r,$$ (4)

where ***r***_*i*_ is the position of the *i*th carbon atom, $r_{i}^{0}$ is its equilibrium position, and the Hessian matrix *M* contains the second derivatives of the elastic energy with respect to atomic coordinates. Here, we take *δ****r*** to be the conformational change from a ligand-free state (1BFE) to a ligand-bound state (1BE9) of the same PDZ domain (Fig 1(a)). This conformational change is central to PDZ function, so its energetic cost has presumably been under selection during evolution. Any other coherent conformational change would also be suitable for our analysis. Note that our aim is not to analyze conformational changes in all their richness, but to provide a minimal concrete example of a relevant additive trait, and to analyze the impact of selection acting on this trait on the associated family of sequences.

To mimic simply the effect of mutation and selection within our toy model, we introduce “mutations” of residues that weaken the spring constants involving their beta carbons by a small fraction *ϵ*. In practice, we take *ϵ* = 0.2. We represent mutations using a sequence $\vec{S}$ with *S*_*l*_ ∈ {0, 1}, where *l* is the residue index: *S*_*l*_ = 0 denotes the reference state, while *S*_*l*_ = 1 implies a mutation (Fig 1(c)). The sequence $\vec{S}$ and the spring network fully determine the Hessian matrix *M*, and thus the energy cost *E* of a conformational change (Eq 4). Note that here $\vec{S}$ is a binary sequence, which represents a simplification compared to real protein sequences $\vec{\alpha }$ where each site can feature 21 states (20 amino acids, plus the alignment gap). We start with the binary model for simplicity, and we then extend our results to a more realistic 21-state model. Note that binary representations of actual protein sequences, with a consensus residue state and a “mutant” state, have proved useful in sector analysis [4], although more recent approaches for diverse protein families have employed full 21-state models [7]. Binary representations are also appropriate to analyze sets of sufficiently close sequences, notably HIV proteins, allowing identification of their sectors [5] and predictions of their fitness landscapes [33].

#### Deformation energy as an additive trait

Focusing on mutations that weakly perturb the elastic properties of a protein, we perform first-order perturbation analysis: *M* = *M*^(0)^ + *ϵM*^(1)^ + *o*(*ϵ*). Using Eq 4 yields *E* = *E*^(0)^ + *ϵE*^(1)^ + *o*(*ϵ*), with *E*^(1)^ = *δ****r***^*T*^*M*^(1)^*δ****r***/2. Both *M*^(1)^ and *E*^(1)^ can be expressed as sums of contributions from individual mutations. We define Δ_*l*_ as the first-order energy cost *ϵE*^(1)^ of a single mutation at site *l* of the sequence. To leading order, the effect of mutations on the energy cost of a deformation reads

$$\delta E=E-E^{\left(0\right)}=\sum_{l=1}^{L}S_{l}\Delta_{l}.$$ (5)

This equation corresponds to the binary-sequence case of the general additive trait defined in Eq 1. Hence, the deformation energy in our toy model of a protein as a sequence-dependent elastic network constitutes a practical example of an additive trait.

Within our functional definition, a protein sector is the set of sites with dominant mutational effects on the trait under selection. The vector $\vec{\Delta }$ of mutational effects for our elastic-network model of the PDZ domain is shown in Fig 1(f). The magnitudes of mutational effects are strongly heterogeneous (Fig. 1 in S1 Appendix). Here, the amino acids with largest effects, which constitute the sector, correspond to those that move most upon ligand binding. (Note that the ligand-binding deformation of PDZ is well-described by one low-frequency normal mode of the elastic network [20]: hence, our sector significantly overlaps with the sites that are most involved in this mode).

How is such a functionally-defined sector reflected in the statistical properties of the sequences that survive evolution? To answer this question, we next analyze sequences obtained by selecting on the trait *δE*. While for concreteness, we use the mutational effects obtained from our elastic model, the analysis is general and applies to any additive trait. Indeed, we later present some examples using synthetically-generated random mutational effect vectors, both binary and more realistic 21-state ones (see below and S1 Appendix).

## Results

### Signature of selection in sequences

For our elastic model of the PDZ domain, the distribution of the additive trait *δE* for random sequences is shown in Fig 1(d). We use the selection process introduced in Eqs 2 and 3 to limit sequences to a narrower distribution of *δE*s, corresponding, e.g., to a preferred ligand-binding affinity. The fitness of a binary sequence $\vec{S}$, a particular case of Eq 3, reads:

$$w\left(\vec{S}\right)=-\frac{\kappa }{2}(\sum_{l=1}^{L}\Delta_{l}S_{l}-\delta E^{*}{)}^{2}.$$ (6)

Here, the selection strength *κ* sets the width of the selection window, and δE* is its center. For all selections, we take $\kappa =10/(\sum_{l}\;\Delta_{l}^{2})$, so that the width of the selection window scales with that of the unselected distribution. We have confirmed that our conclusions are robust to varying selection strength, provided $\kappa \sum_{l}\;\Delta_{l}^{2}\gg 1$ (see Fig. 3 in S1 Appendix).

Although mutations have additive effects on the trait *δE*, the nonlinearities involved in fitness and selection give rise to correlations among sites. For instance, if δE* = 0 and if Δ_*l*_ < 0 for all *l*, as in Fig 1, a mutation at a site with large |Δ_*l*_| will decrease the likelihood of additional mutations at all other sites with large |Δ_*l*_|.

Previous approaches to identifying sectors from real protein sequences have relied on modified forms of Principal Component Analysis (PCA). So we begin by asking: can PCA identify sectors in our physical model? PCA corresponds to diagonalizing the covariance matrix *C* of sequences: it identifies the principal components (eigenvectors) ${\vec{\nu }}^{\left(j\right)}$ associated with progressively smaller variances (eigenvalues) λ^(*j*)^. We introduce 〈⋅〉_*_ to denote ensemble averages over the selectively weighted sequences, reserving 〈⋅〉 for averages over the unselected ensemble. The mutant fraction at site *l* in the selected ensemble is ${\langle S_{l}\rangle }_{*}=\sum_{\vec{S}}S_{l}P\left(\vec{S}\right)$, and the covariance matrix *C* reads

$$C_{ll^{\prime }}={\langle \left(S_{l}-{\langle S_{l}\rangle }_{*}\right)\cdot \left(S_{l^{\prime }}-{\langle S_{l^{\prime }}\rangle }_{*}\right)\rangle }_{*}.$$ (7)

To test the ability of PCA to identify a functional sector, we employed the selection window shown in orange in Fig 1(d). The resulting eigenvalues are shown in Fig 1(e). One sees outliers. In particular, why is the last eigenvalue so low? Due to the narrow selection window, according to Eq 6 the highly-weighted sequences satisfy $\sum_{l}\;S_{l}\Delta_{l}=\vec{S}\cdot \vec{\Delta }\approx \delta E^{*}$. This means that in the *L*-dimensional sequence space, the data points for the highly-weighted sequences lie in a narrow region around a plane perpendicular to $\vec{\Delta }$ (Fig 1(g)). Hence, the data has exceptionally small variance in this direction, leading to a particularly small eigenvalue of *C*. Moreover, the corresponding last principal component ${\vec{\nu }}^{\left(L\right)}$ points in the direction with the smallest variance and is consequently parallel to $\vec{\Delta }$ (Fig 1(f)). Formally, in Eq 6, $\vec{\Delta }$ appears in a quadratic coupling term where it plays the part of a repulsive pattern in a generalized Hopfield model [34, 35]: alone, such a term would penalize sequences aligned with $\vec{\Delta }$. But here, $\vec{\Delta }$ also appears in a term linear in $\vec{S}$ and as a result Eq 6 penalizes sequences that fail to have the selected projection onto $\vec{\Delta }$.

In this example, the last principal component accurately recovers the functional sector corresponding to the largest elements of the mutational-effect vector $\vec{\Delta }$. More generally, to quantify the recovery of $\vec{\Delta }$ by a given vector $\vec{\nu }$, we introduce

$$\text{Recovery}=\frac{\sum_{l}\left|\nu_{l}\Delta_{l}\right|}{\sqrt{\sum_{l}\nu_{l}^{2}}\sqrt{\sum_{l}\Delta_{l}^{2}}},$$ (8)

which is nonnegative, has a random expectation of $\left(\sqrt{2/\pi L}\right)\sum_{l}\left|\Delta_{l}\right|/\sqrt{\sum_{l}\;\Delta_{l}^{2}}$ for *L* ≫ 1 (S1 Appendix), and saturates at 1 (including the case of parallel vectors). For our test case, Fig 1(h) shows Recovery for all principal components. The last one features the highest Recovery, almost 1, confirming that it carries substantial information about $\vec{\Delta }$. The second-to-last principal component and the first two also provide a value of Recovery substantially above random expectation. Outlier eigenvalues arise from the sector, and accordingly, we find that the number of modes with high Recovery often corresponds to the number of sites with strong mutational effects. A more formal analysis of this effect will be an interesting topic for further study.

In our model, $\vec{\Delta }$ is fundamentally a direction of *small variance*. So why do the first principal components also carry information about $\vec{\Delta }$? Qualitatively, when variance is decreased in one direction due to a repulsive pattern $\vec{\Delta }$, variance tends to increase in orthogonal directions involving the same sites. To illustrate this effect, let *L* = 3 and $\vec{\Delta }=\left(-1,1,0\right)$, and consider the sequences $\vec{S}$ satisfying $\vec{\Delta }\cdot \vec{S}=0$ (namely (0, 0, 0); (1, 1, 0); (0, 0, 1); (1, 1, 1)). The last principal component is $\vec{\Delta }$, with zero variance, and the first principal component is (1, 1, 0): Recovery is 1 for both of them. This selection conserves the trace of the covariance matrix (i.e. the total variance), so that decreasing the variance along $\vec{\Delta }=\left(-1,1,0\right)$ necessarily increases it along (1, 1, 0). This simple example provides an intuitive understanding of why the large-eigenvalue modes of the covariance matrix also carry information about $\vec{\Delta }$.

It is worth remarking that Eq 6 is a particular case of a general fitness function with one- and two-body terms (known as fields and couplings in Ising or Potts models in physics). Here, the values of these one- and two-body terms are constrained by their expressions in terms of $\vec{\Delta }$. In practice, several traits might be selected simultaneously (see below), yielding more independent terms among the fields and couplings. More generally, such one- and two-body descriptions have been very successfully employed via Direct Coupling Analysis (DCA) to identify strongly coupled residues that are in contact within a folded protein [36–38], to investigate folding [39], and to predict fitness [33, 40–45] and conformational changes [46, 47], as well as protein-protein interactions [48, 49]. A complete model of protein covariation in nature should necessarily incorporate both the collective modes described here and the strongly coupled residue pairs which are the focus of DCA.

### ICOD method

**Fig 2:**
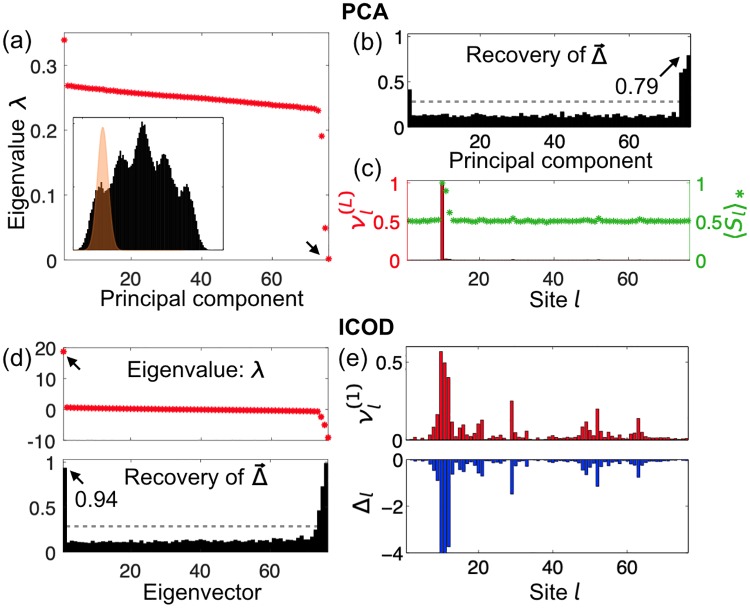
Recovery of mutational-effect vector $\vec{\Delta }$ from sequence analysis in the case of strongly biased selection. (a-c) Principal Component Analysis (PCA) performs poorly due to strong conservation at some sites of large mutational effect. (a) Eigenvalues of covariance matrix obtained for strongly biased selection around $\delta E_{\text{biased}}^{*}$ (inset, orange window) for same model proteins as in Fig 1. (b) Recovery of $\vec{\Delta }$ for all principal components. (c) Last principal component $\nu_{l}^{\left(L\right)}$ (red) and average mutant fraction 〈*S*_*l*_〉_*_ (green) at site *l*. (d-e) The ICOD method performs robustly. (d) Eigenvalues of ${\tilde{C}}_{ll^{\prime }}^{-1}$ (Eq 10) (upper) and Recovery of $\vec{\Delta }$ for all eigenvectors (lower). (e) Leading eigenvector $\nu_{l}^{\left(1\right)}$ (upper) and mutational effect Δ_*l*_ at site *l* (lower, same as in Fig 1(f)). Gray dashed lines in (b, d): random expectation of Recovery (S1 Appendix).

An important concern is whether the last principal component is robust to small and/or noisy datasets. Indeed, other directions of small variance can appear in the data. As a second example, we applied a different selection window, centered in the tail of the distribution of *δE*s from our elastic model of the PDZ domain (Fig 2(a), inset). This biased selection generates strong conservation, 〈*S*_*l*_〉_*_ ≈ 1, for some sites with significant mutational effects. Extreme conservation at one site now dictates the last principal component, and disrupts PCA-based recovery of $\vec{\Delta }$ (Fig 2(a) and 2(b)).

To overcome this difficulty, we developed a more robust approach that relies on inverting the covariance matrix. Previously, the inverse covariance matrix was successfully employed in Direct Coupling Analysis (DCA) to identify strongly coupled residues that are in contact within a folded protein [36–38]. The fitness in our model (Eq 6) involves one and two-body interaction terms, and constitutes a particular case of the DCA Hamiltonian (S1 Appendix). A small-coupling approximation [37, 38, 50, 51] (S1 Appendix) gives

$$C_{ll^{\prime }}^{-1}\approx (1-\delta_{ll^{\prime }})\;\kappa \Delta_{l}\Delta_{l^{\prime }}+\delta_{ll^{\prime }}(\frac{1}{P_{l}}+\frac{1}{1-P_{l}}),$$ (9)

where *P*_*l*_ denotes the probability that site *l* is mutated. Since we are interested in extracting $\vec{\Delta }$, we can simply set to zero the diagonal elements of *C*^−1^, which are dominated by conservation effects, to obtain a new matrix

$${\tilde{C}}_{ll^{\prime }}^{-1}\approx \left(1-\delta_{ll^{\prime }}\right)\kappa \Delta_{l}\Delta_{l^{\prime }}.$$ (10)

The first eigenvector of ${\tilde{C}}^{-1}$ (associated with its largest eigenvalue) should accurately report $\vec{\Delta }$ since, except for its zero diagonal, ${\tilde{C}}^{-1}$ is proportional to the outer product $\vec{\Delta }⊗\vec{\Delta }$. We call this approach the *Inverse Covariance Off-Diagonal* (ICOD) method. As shown in Fig 2(d) and 2(e), ICOD overcomes the difficulty experienced by PCA for biased selection, while performing equally well as PCA for unbiased selection (Fig. 2 in S1 Appendix). Removing the diagonal elements of *C*^−1^ before diagonalizing is crucial: otherwise, the first eigenvector of *C*^−1^ is the same as the last eigenvector of *C* and suffers from the same shortcomings for strong conservation. Here too, eigenvectors associated to both small and large eigenvalues contain information about $\vec{\Delta }$ (Fig 2(b) and 2(d)).

### Selection on multiple traits

An important challenge in sector analysis is distinguishing multiple, independently evolving sectors [4, 7, 52]. We can readily generalize our fitness function (Eqs 3 and 6) to allow for selection on multiple additive traits:

$$w\left(\vec{S}\right)=-\sum_{i=1}^{N}\frac{\kappa_{i}}{2}(\sum_{l=1}^{L}\Delta_{i,l}S_{l}-T_{i}^{*}{)}^{2},$$ (11)

where *N* is the number of distinct additive traits $T_{i}\left(\vec{S}\right)=\sum_{l}\;\Delta_{i,l}S_{l}$ under selection, ${\vec{\Delta }}_{i}$ is the vector of mutational effects on trait *T*_*i*_, *κ*_*i*_ is the strength of selection on this trait, and $T_{i}^{*}$ is the associated selection bias. For example, ${\vec{\Delta }}_{1}$ might measure how mutations change a protein’s binding affinity, while ${\vec{\Delta }}_{2}$ might be related to its thermal stability, etc.

In Fig. 5 in S1 Appendix, we consider selection on two distinct additive traits, using synthetically-generated random mutational-effect vectors ${\vec{\Delta }}_{1}$ and ${\vec{\Delta }}_{2}$ (S1 Appendix). Note that these mutational effects are thus unrelated to our toy model of protein elastic deformations: as stated above, our approach holds for any additive trait under selection. ICOD then yields *two* large outlier eigenvalues of the modified inverse covariance matrix ${\tilde{C}}^{-1}$. The associated eigenvectors accurately recover both ${\vec{\Delta }}_{1}$ and ${\vec{\Delta }}_{2}$, after a final step of Independent Component Analysis (ICA) [7, 53, 54] that successfully disentangles the contributions coming from the two constraints (see S1 Appendix).

### Performance in sector recovery

**Fig 3:**
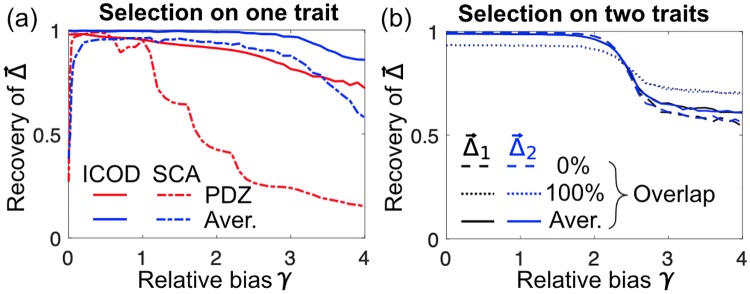
Average recovery of mutational-effect vectors $\vec{\Delta }$ as a function of relative selection bias $\gamma \equiv \left(T^{*}-\langle T\rangle \right)/\sqrt{\langle {\left(T-\langle T\rangle \right)}^{2}\rangle }$ on the selected additive trait *T*. (a) Selection on a single trait. Different $\vec{\Delta }\text{s}$ are used to generate sequence ensembles: the elastic-network $\vec{\Delta }$ from Fig 1 (red); synthetic $\vec{\Delta }\text{s}$ (S1 Appendix) with number of sites of large mutational effect (sector sites) ranging from 1 to 100, for sequences of length *L* = 100 (blue). Recovery is shown for ICOD (solid curves) and for SCA [4, 7] (dashed curves). (b) Selection on two distinct traits. Different pairs of synthetic $\vec{\Delta }\text{s}$ (S1 Appendix) are used to generate sequence ensembles (with *L* = 100): “0%” indicates two non-overlapping sectors, each with 20 sites; “100%” indicates two fully overlapping sectors, each with 100 sites; “Aver.” indicates average Recovery over 100 cases of double selection, where the single-sector size increases from 1 to 100, and the overlap correspondingly increases from 0 to 100. ICA was applied to improve Recovery (S1 Appendix).

We further tested the performance of ICOD by systematically varying the selection bias, both for our toy model of PDZ elastic deformations and for more general synthetically-generated random mutational-effect vectors (Fig 3). ICOD achieves high Recovery of these various mutational-effect vectors for both single and double selection over a broad range of selection biases T*, albeit performance falls off in the limit of extreme bias.

How does ICOD compare with other approaches to identifying sectors? We compared the performance of ICOD with Statistical Coupling Analysis (SCA), the original PCA-based method [4, 7]. In SCA, the covariance matrix *C* is reweighted by a site-specific conservation factor *ϕ*_*l*_, the absolute value is taken, ${\tilde{C}}_{ll^{\prime }}^{\left(\text{SCA}\right)}=\left|\phi_{l}C_{ll^{\prime }}\phi_{l^{\prime }}\right|$, and sectors are identified from the leading eigenvectors of ${\tilde{C}}^{\left(\text{SCA}\right)}$. We therefore tested the ability of the first eigenvector of ${\tilde{C}}^{\left(\text{SCA}\right)}$ to recover $\vec{\Delta }$ for a single selection. We found that the square root of the elements of the first SCA eigenvector can provide high Recovery of $\vec{\Delta }$ (Fig 3, and Figs. 13, 14 in S1 Appendix). However, the performance of SCA relies on conservation through *ϕ*_*l*_, and it has been shown that residue conservation actually dominates sector identification by SCA in certain proteins [52]. Consequently, for unbiased selection, SCA breaks down (Fig 3(a), dashed curves) and cannot identify sector sites (Fig. 17 in S1 Appendix). ICOD does not suffer from such shortcomings, and performs well over a large range of selection biases. Note that in SCA, only the top eigenvectors of ${\tilde{C}}^{\left(\text{SCA}\right)}$ convey information about sectors (Figs. 13, 15 in S1 Appendix).

We also compared ICOD with another PCA-based approach [34], which employs an inference method specific to the generalized Hopfield model, and should thus be well adapted to identifying sectors within our physical model (Eq 6). Overall, this specialized approach performs similarly to ICOD, being slightly better for very localized sectors, but less robust than ICOD for strong selective biases and small datasets (S1 Appendix). Exactly as for PCA and ICOD, within this method, the top Recovery is obtained for the bottom eigenvector of the (modified) covariance matrix, consistent with $\vec{\Delta }$ in our model being a repulsive pattern [34], but large Recoveries are also obtained for the top eigenvectors (Fig. 18 in S1 Appendix).

### Robustness to different forms of selection

**Fig 4:**
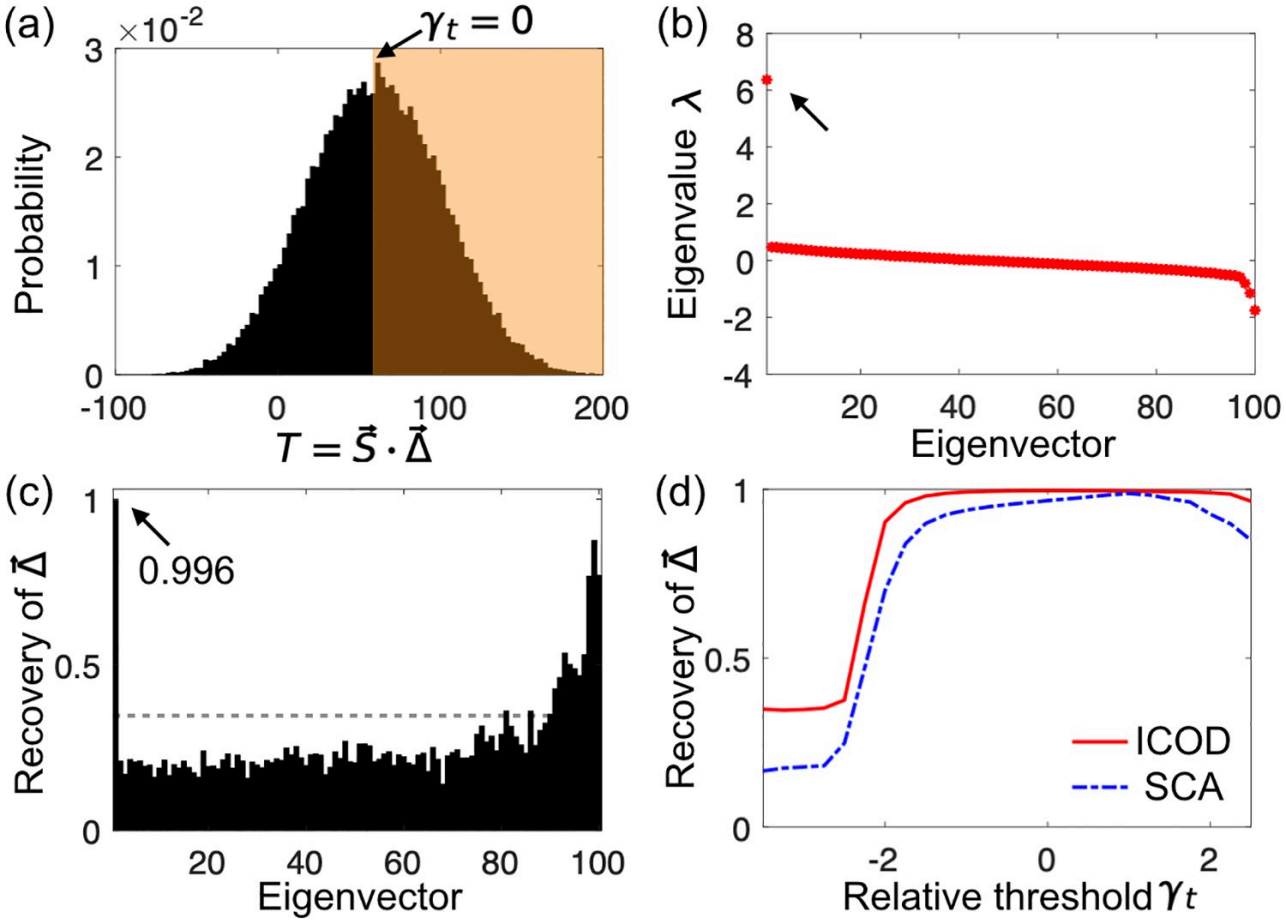
Identification of sectors that result from threshold-based selection. (a) Histogram of the additive trait $T\left(\vec{S}\right)=\vec{S}\cdot \vec{\Delta }$ for randomly sampled sequences where 0 and 1 are equally likely at each site. Sequence length is *L* = 100, mutational effects are synthetically generated with 20 sector sites (see S1 Appendix). Sequences are selected if they have a trait value $T\left(\vec{S}\right)>T_{t}$ (orange shaded region). Selection is shown for *T*_*t*_ = 〈*T*〉, or equivalently *γ*_*t*_ = 0, in terms of the relative threshold $\gamma_{t}\equiv \left(T_{t}-\langle T\rangle \right)/\sqrt{\langle {\left(T-\langle T\rangle \right)}^{2}\rangle }$. (b) Eigenvalues of the ICOD-modified inverse covariance matrix ${\tilde{C}}^{-1}$ (Eq 10) of the selected sequences for *γ*_*t*_ = 0. (c) Recovery of $\vec{\Delta }$ for all eigenvectors of ${\tilde{C}}^{-1}$ for *γ*_*t*_ = 0. Gray dashed line: random expectation of Recovery. (d) Recovery of $\vec{\Delta }$ for ICOD and for SCA as functions of the relative selection threshold *γ*_*t*_. The data in (d) is averaged over 100 realizations of $\vec{\Delta }$.

To assess the robustness of functional sectors to selections different from the simple Gaussian selection window of Eqs 2 and 3, we selected sequences with an additive trait *T* above a threshold *T*_*t*_, and varied this threshold. For instance, a fluorescent protein might be selected to be fluorescent enough, which could be modeled by requiring that (a nonlinear transform of) an additive trait be sufficiently large [10]. As shown in Fig 4, the corresponding sectors are identified by ICOD as well as those resulting from our initial Gaussian selection window. In Fig 4(d), we show the performance of both ICOD and SCA at recovering sectors arising from selection with a threshold. Consistent with previous results (see Fig 3), we find that ICOD is more robust than SCA to extreme selections. We also successfully applied ICOD to other forms of selection: Fig. 8 in S1 Appendix shows the case of a quartic fitness function replacing the initial quadratic one (Eq 3) in the Boltzmann distribution (Eq 2) and Fig. 9 in S1 Appendix shows the case of a rectangular selection window (S1 Appendix). These results demonstrate the robustness of functional sectors, and of ICOD, to different plausible forms of selection.

### Extension to 21-state sequences and to natural sequences

So far, we have considered binary sequences, with only one type of mutation with respect to the reference state. In the S1 Appendix, we demonstrate that our formalism, including the ICOD method, extends to mutations among *q* different states. The case *q* = 21, which includes the 20 different amino-acid types plus the alignment gap is the relevant one for real proteins. The single-site mutational effects Δ_*l*_ are then replaced by state-specific mutational effects Δ_*l*_(*α*_*l*_) with *α*_*l*_ ∈ {1, …, 21} (see Eq 1). Fig. 10 in S1 Appendix shows that the generalized version of ICOD performs very well on synthetic data generated for the case *q* = 21. We further demonstrate that sector identification is robust to gauge changes (reference changes) and to the use of pseudocounts (S1 Appendix).

**Fig 5:**
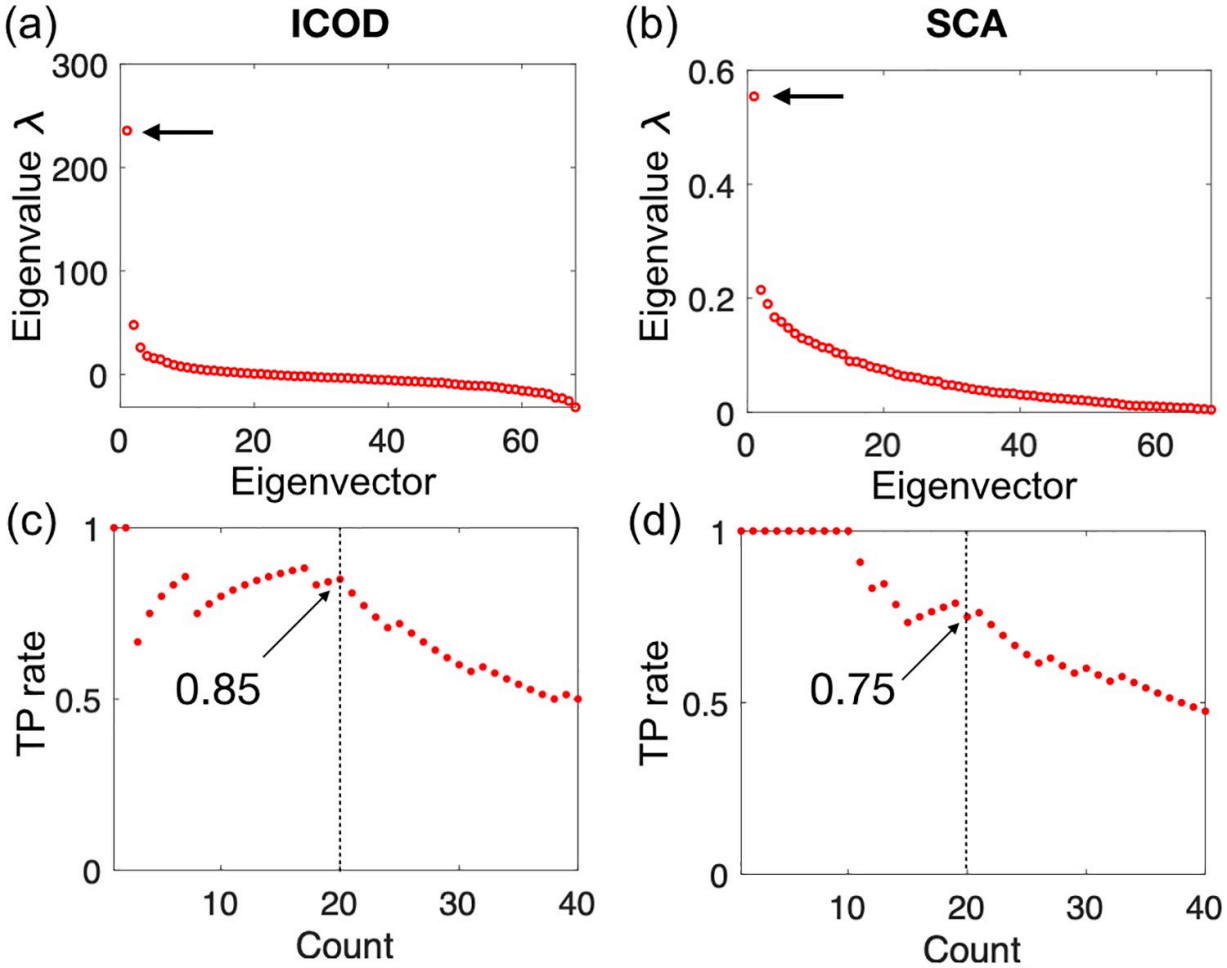
Performance of ICOD and SCA at predicting the 20 sites with largest experimentally-determined mutational effects in a PDZ domain. (a) Eigenvalues of the compressed ICOD-modified inverse covariance matrix ${\tilde{C}}^{-1}$ (S1 Appendix). (b) Eigenvalues of the SCA matrix. (c) True Positive (TP) rates obtained by taking the first eigenvector ${\vec{\nu }}^{\left(1\right)}$ from the compressed ICOD-modified inverse covariance matrix, generating a ranked list of sites of descending magnitudes of the components $\left|\right|\nu_{l}^{\left(1\right)}\left|\right|$ of this eigenvector at each site *l* (S1 Appendix), and computing the fraction of the top sites in this predicted ordering that are also among the 20 experimentally most important sites [6]. Results are shown versus the number of top predicted sites (“count”). (d) TP rates from SCA, computed as in panel (c). In panels (c) and (d), the TP rate values obtained for the top 20 predicted sites are indicated by arrows. In all panels, a pseudocount ratio Λ = 0.02 was used, and sites with more than 15% gap state were discarded (see S1 Appendix for details).

While the main purpose of this article is to propose an operational definition of functional protein sectors and to understand how they can arise, an interesting next question will be to investigate what ICOD can teach us about real data. As a first step in this direction, we applied ICOD to a multiple sequence alignment of PDZ domains. In this analysis, we employed a complete description with *q* = 21, but we compressed the ICOD-modified inverse matrix using the Frobenius norm to focus on overall (and not residue-specific) mutational effects (see S1 Appendix for details). As shown in Fig 5(a) and 5(b), both ICOD and SCA identify one strong outlying large eigenvalue, thus confirming that PDZ has only one sector [6]. Recall that due to the inversion step, the largest eigenvalue in ICOD is related to the mode with smallest variance, whose importance was demonstrated above. Furthermore, as seen in Fig 5(c) and 5(d), both methods correctly predict the majority of residues found experimentally to have important mutational effects on ligand binding to the PDZ domain shown in Fig 1(a) [6]. For instance, over the 20 top sites identified by ICOD (resp. SCA), we find that 85% (resp. 75%) of them are also among the 20 experimentally most important sites. Note that for SCA, we recover the result from Ref. [6]. The performance of ICOD is robust to varying the cutoff for removal of sites with a large proportion of gaps (see Fig. 21 in S1 Appendix), but notably less robust than SCA to pseudocount variation (see Fig. 22 in S1 Appendix).

Importantly, both ICOD and SCA perform much better than random expectation, which is 29%. Hence, both of these methods can be useful to identify functionally important sites. The slightly greater robustness of SCA to pseudocounts on this particular dataset (see Fig. 22 in S1 Appendix) might come from the fact that many of the experimentally-identified functionally important sites in the PDZ domain are strongly conserved [52], which makes the conservation reweighting step in SCA advantageous. Since residue conservation alone is able to predict most of the experimentally important PDZ sites [52], we also compared conservation to SCA and ICOD: ranking sites by conservation (employing the conservation score of Ref. [7], see S1 Appendix) indeed identifies 70% of the top 20 experimentally-determined sites with important mutational effects. Interestingly, ICOD scores are slightly more strongly correlated with conservation than SCA scores are correlated with conservation (see Fig. 23 in S1 Appendix), despite the fact that conservation is explicitly used in SCA and not in ICOD.

Overall, this preliminary application to real data highlights the ability of ICOD to identify functionally related amino acids in a principled way that only relies on covariance. We emphasize that the main goal of this paper is to provide insight into the possible physical origins of sectors, and into the statistical signatures of these physical sectors in sequence data. A more extensive application of ICOD and related methods to real sequence data will be the subject of future work.

## Discussion

We have demonstrated how sectors of collectively correlated amino acids can arise from evolutionary constraints on functional properties of proteins. Our model is very general, as it only relies on the functional property under any of various forms of selection being described by an underlying additive trait, which has proven to be valid in many relevant situations [8–10, 13].

We showed that the primary signature of functional selection acting on sequences lies in the small-eigenvalue modes of the covariance matrix. In contrast, sectors are usually identified from the large-eigenvalue modes of the SCA matrix [4, 7]. This is not in contradiction with our results because, as we showed, signatures of our functional sectors are often also found in large-eigenvalue modes of the covariance matrix. Besides, the construction of the SCA matrix from the covariance matrix involves reweighting by conservation and taking an absolute value or a norm [4, 7], which can substantially modify its eigenvectors, eigenvalues, and their order. Conservation is certainly important in real proteins, especially in the presence of phylogeny; indeed, the SCA matrix, which includes both conservation and covariance, was recently found to capture well experimentally-measured epistasis with respect to the free energy of PDZ ligand binding [55]. However, the fundamental link we propose between functional sectors and small-eigenvalue modes of the covariance matrix is important, since large-eigenvalue modes of the covariance matrix also contain confounding information about subfamily-specific residues [56] and phylogeny [57], and consistently, some sectors identified by SCA have been found to reflect evolutionary history rather than function [4]. Interestingly, the small-eigenvalue modes are also the ones that contain most information about structural contacts in real proteins [35]. Hence, our results help explain previously observed correlations between sectors and contacts, e.g. the fact that contacts are overrepresented within a sector but not across sectors [58].

We introduced a principled method to detect functional sectors from sequence data, based on the primary signature of these sectors in the small-eigenvalue modes of the covariance matrix. We further demonstrated the robustness of our approach to the existence of multiple traits simultaneously under selection, to various forms of selection, and to data-specific questions such as reference choices and pseudocounts.

Importantly, our modeling approach allowed us to focus on functional selection alone, in the absence of historical contingency and of specific structural constraints, thus yielding insights complementary to purely data-driven methods. The collective modes investigated here are just one source of residue-residue correlations. Next, it will be interesting to study the intriguing interplay between functional sectors, phylogeny, and contacts, and to apply our methods to multiple protein families. Our results shed light on an aspect of the protein sequence-function relationship and open new directions in protein sequence analysis, with implications in synthetic biology, building toward function-driven protein design.

## Supporting information

S1 Appendix Methodological details and further results. In S1 Appendix, we present additional details about our model and methods, as well as additional results.
(PDF)
