# Supplementary material for: Revealing evolutionary constraints on proteins through sequence analysis
Source: PLoS Comput Biol. 2019 Apr 24;15(4):e1007010. doi: 10.1371/journal.pcbi.1007010 (PMC6502352; doi:10.1371/journal.pcbi.1007010)
Supplement: S1 Appendix — In S1 Appendix, we present additional details about our model and methods, as well as additional results. (PDF) [file pcbi.1007010.s001.pdf]

Revealing evolutionary constraints on proteins through sequence analysis - S1 Appendix

Shou-Wen Wang[1,2,3◐¤], Anne-Florence Bitbol[4◐*], Ned S. Wingreen[3,5*]

1 Department of Engineering Physics, Tsinghua University, Beijing, 100086, China
2 Beijing Computational Science Research Center, Beijing, 100094, China
3 Lewis-Sigler Institute for Integrative Genomics, Princeton University, Princeton, NJ 08544, USA
4 Sorbonne Université, CNRS, Laboratoire Jean Perrin (UMR 8237), F-75005 Paris, France
5 Department of Molecular Biology, Princeton University, Princeton, NJ 08544, USA

◐These authors contributed equally to this work.
¤Current Address: Department of Systems Biology, Harvard Medical School, Boston, MA 02115, USA.
* anne-florence.bitbol@sorbonne-universite.fr (A.-F. B.), wingreen@princeton.edu (N. S. W.)

Contents

1 Supplemental results for elastic network model of PDZ domain 2

2 Recovery by a random vector 2

3 Inverse covariance matrix of our sequence ensembles 3
3.1 Binary sequences . . . 4
3.1.1 From a sector model for binary sequences to an Ising model . . . 4
3.1.2 First-order small-coupling expansion . . . 4
3.1.3 Application to our sector model . . . 5
3.2 Sequences with q possible states at each site . . . 6
3.2.1 From a sector model to a Potts model for sequences . . . 6
3.2.2 First-order small-coupling expansion . . . 6
3.2.3 Application to our sector model . . . 7
3.2.4 Selection on multiple traits . . . 7

4 Robustness of functional sectors and of ICOD 8
4.1 Robustness of ICOD to selection bias, selection strength, and multiple selections . . . 8
4.2 Robustness of functional sectors to different forms of selection . . . 10
4.3 Multiple states per site and alternative gauge choice . . . 12
4.4 Pseudocounts . . . 14

5 Performance of SCA 15
5.1 Analytical estimates for $\langle S_l \rangle_*$ and $C_{ll'}$ for a single selection with binary sequences . . . 15
5.2 Analysis of the SCA method . . . 16
5.3 Comparison between ICOD and SCA . . . 18

6 Performance of a method based on the generalized Hopfield model 19

7 Application of ICOD to a multiple sequence alignment of PDZ domains 22

1 Supplemental results for elastic network model of PDZ domain

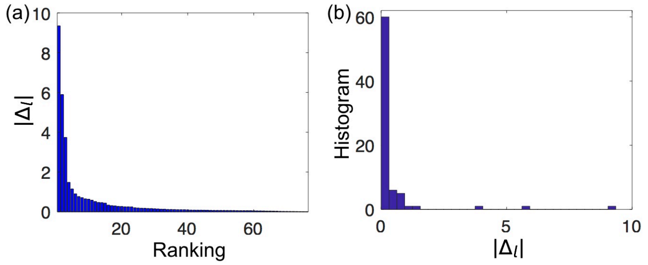

Fig 1. Magnitude of single-site mutational effects Δ_l for the PDZ domain conformational change from Fig. 1 of the main text. (a) Magnitudes by rank. (b) Histogram of magnitudes. According to our definition, the sites of large magnitude constitute "sector" sites with respect to selection on the energy cost of this conformational change, while all others are "non-sector" sites.

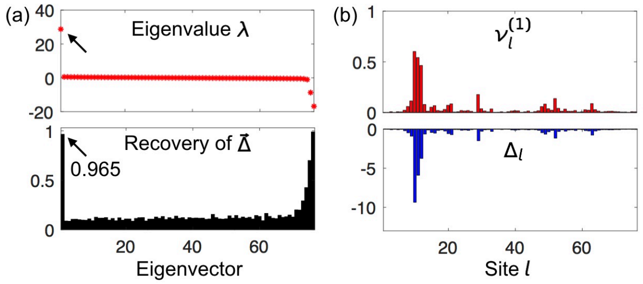

Fig 2. Performance of ICOD for the selected sequence ensemble from Fig. 1 of the main text. (a) Eigenvalues for ICOD method (upper) and Recovery of $\vec{\Delta}$ for all eigenvectors (lower). (b) Leading eigenvector $\nu_l^{(1)}$ (upper) and mutational effect Δ_l at site l (lower, same as in Fig. 1(f) of the main text). The excellent performance of ICOD on this unbiased ensemble of sequences supports the general applicability of the ICOD method to both biased and unbiased sequence ensembles.

2 Recovery by a random vector

Here, we calculate the random expectation of the Recovery of the mutational-effect vector $\vec{\Delta}$ by a generic other vector $\vec{\nu}$, in order to establish a null model to which to compare. For a binary sequence, Recovery, as defined in Eq. 8 of the main text, can be expressed as

$$\text{Recovery} = \vec{\Delta}' \cdot \vec{\nu}' = \sum_{l=1}^{L} \Delta'_l \nu'_l, \tag{1}$$

with $\Delta'_l = |\Delta_l| / \sqrt{\sum_l \Delta_l^2}$ and $\nu'_l = |\nu_l| / \sqrt{\sum_l \nu_l^2}$. As before, L denotes the length of the sequence and hence the number of components of $\vec{\Delta}$ and $\vec{\nu}$. As $\vec{\nu'}$ is a normalized L-dimensional vector, its components can be expressed in L-dimensional spherical coordinates using $L-1$ angles θ_i:

$$\nu'_l = \left(\prod_{i=1}^{l-1} \sin\theta_i \right) \cos\theta_l \qquad \forall l \in \{1, \ldots, L-1\}, \tag{2}$$

$$\nu'_L = \prod_{i=1}^{L-1} \sin\theta_i , \tag{3}$$

where $\theta_i \in [0, \pi/2]$ for all $i \in \{1, \cdots, L\}$, because all components of $\vec{\nu'}$ are nonnegative. Note that we employ the usual convention that empty products are equal to one: Eq. 2 yields $\nu'_1 = \cos\theta_1$.

The average Recovery for a random vector $\vec{\nu'}$ with an orientation uniformly distributed in the L-dimensional sphere reads:

$$\langle \text{Recovery} \rangle = \frac{\int_\Omega d\Omega \ \sum_l \Delta'_l \nu'_l}{\int_\Omega d\Omega} = \frac{\sum_l \Delta'_l \, I_l}{\int_\Omega d\Omega} \,, \tag{4}$$

where the angular element is $d\Omega = \prod_{i=1}^{L-1} d\theta_i \ \sin^{L-i-1}(\theta_i)$, the integration domain is $\Omega = [0, \pi/2]^{L-1}$, and we have introduced $I_l = \int_\Omega d\Omega \, \nu'_l$. Using Eq. 2, we obtain for $1 \leq l \leq L-1$

$$I_l = \int_\Omega d\Omega \, \nu'_l = \left(\prod_{i=1}^{l-1} \int_0^{\pi/2} d\theta_i \ \sin^{L-i}(\theta_i) \right) \left(\int_0^{\pi/2} d\theta_l \ \sin^{L-l-1}(\theta_l) \cos(\theta_l) \right) \left(\prod_{i=l+1}^{L-1} \int_0^{\pi/2} d\theta_i \ \sin^{L-i-1}(\theta_i) \right) \,, \tag{5}$$

and similarly, Eq. 3 yields

$$I_L = \int_\Omega d\Omega \, \nu'_L = \prod_{i=1}^{L-1} \int_0^{\pi/2} d\theta_i \ \sin^{L-i}(\theta_i) \, . \tag{6}$$

Using the following results valid for $n > -1$:

$$\int_0^{\pi/2} d\theta \ \sin^n(\theta) = \frac{\sqrt{\pi}}{2} \frac{\Gamma\left(\frac{1+n}{2}\right)}{\Gamma\left(\frac{n+2}{2}\right)} \, ; \quad \int_0^{\pi/2} d\theta \ \sin^n(\theta) \cos(\theta) = \frac{1}{n+1} \,, \tag{7}$$

where Γ denotes the Euler Gamma function, which satisfies $\Gamma(x+1) = x \, \Gamma(x)$ for all x, we obtain for $1 \leq l \leq L$:

$$I_l = \frac{\pi^{(L-1)/2}}{2^{L-1} \, \Gamma\left(\frac{L+1}{2}\right)}, \tag{8}$$

which is independent of l. Besides,

$$\int_\Omega d\Omega = \frac{\pi^{L/2}}{2^{L-1} \, \Gamma(L/2)}. \tag{9}$$

Combining Eq. 4 with Eqs. 8 and 9 finally yields

$$\langle \text{Recovery} \rangle = \frac{\sum_l \Delta'_l \, I_l}{\int_\Omega d\Omega} = \frac{\Gamma\left(L/2\right)}{\sqrt{\pi} \, \Gamma\left(\frac{L+1}{2}\right)} \sum_l \Delta'_l = \frac{\Gamma\left(L/2\right)}{\sqrt{\pi} \, \Gamma\left(\frac{L+1}{2}\right)} \frac{\sum_l |\Delta_l|}{\sqrt{\sum_l \Delta_l^2}}. \tag{10}$$

In particular, in the relevant regime $L \gg 1$, an asymptotic expansion of Γ yields:

$$\langle \text{Recovery} \rangle \approx \sqrt{\frac{2}{\pi L}} \, \frac{\sum_l |\Delta_l|}{\sqrt{\sum_l \Delta_l^2}}. \tag{11}$$

The maximum expectation of Recovery is obtained when all components of $\vec{\Delta}$, i.e. all mutational effects, are identical:

$$\langle \text{Recovery} \rangle_{\max} = \sqrt{\frac{2}{\pi}} \approx 0.798. \tag{12}$$

Conversely, the average Recovery becomes minimal when only one component of $\vec{\Delta}$ is nonzero, which constitutes the limit of the case where the mutational effect at one site is dominant:

$$\langle \text{Recovery} \rangle_{\min} = \sqrt{\frac{2}{\pi L}}, \tag{13}$$

which approaches zero in the limit $L \to \infty$.

3 Inverse covariance matrix of our sequence ensembles

Here, we present a derivation of the small-coupling approximation of the inverse covariance matrix for our artificially-generated sequence ensembles. In this small-coupling limit, the inverse covariance matrix provides an estimate of the energetic couplings used to generate the data. More generally, deducing energetic parameters from observed statistics is a well-known inference problem, also known as an inverse problem. Two-body energetic couplings can be

inferred from the one and two-body frequencies observed in the data, using a standard maximum entropy approach. However, the exact calculation of the energetic terms is difficult, and various approximations have been developed. Following Refs [1,2], we use the mean-field or small-coupling approximation, which was introduced in Ref. [3] for the Ising spin-glass model. For the sake of completeness, we now review the main steps of the calculation, which follow Ref. [2]. Note that we do not use inference methods specific to low-rank coupling matrices [4,5] because we wish to retain generality, with the application to real sequence data in mind.

We begin with the case of binary sequences, which is discussed in the main text. Following that, we generalize to cases where more than two states are allowed at each site, such as the 21 possible states for real protein sequence (20 amino acids plus gap).

3.1 Binary sequences

We begin by deriving Eq. 9 from the main text, which provides an approximation for the inverse covariance matrix of the ensembles of our binary artificial sequences. Each sequence $\vec{S}$ is such that $S_l \in \{0,1\}$ for each site l with $1 \leq l \leq L$, where L is the length of the sequence.

3.1.1 From a sector model for binary sequences to an Ising model

Recall the fitness w of a binary sequence $\vec{S}$ under selection for trait T to be close to T^* (Eq. 6 in the main text):

$$w(\vec{S}) = -\frac{\kappa}{2}\left(T(\vec{S}) - T^*\right)^2 = -\frac{\kappa}{2}\left(\sum_l \Delta_l S_l - T^*\right)^2 . \tag{14}$$

We introduce $s_l = 2S_l - 1$: it is an "Ising spin" variable ($S_l = 0 \Leftrightarrow s_l = -1$ and $S_l = 1 \Leftrightarrow s_l = 1$). The fitness in Eq. 14 can be rewritten as

$$w(\vec{s}) = -\frac{\kappa}{2}\left(\sum_l D_l s_l - \alpha\right)^2 , \tag{15}$$

with $D_l = \Delta_l/2$ and $\alpha = T^* - \sum_l D_l$. Expanding yields

$$w(\vec{s}) = -\frac{\kappa}{2}\left(\sum_{l\neq p} D_l D_p s_l s_p + \sum_l D_l^2 - 2\alpha \sum_l D_l s_l + \alpha^2\right) , \tag{16}$$

where we have used the fact that $s_l^2 = 1$. The second term and the last term in Eq. 16 are both constants, and therefore our fitness is equivalent to

$$w(\vec{s}) = -\frac{\kappa}{2}\left(\sum_{l\neq p} D_l D_p s_l s_p - 2\alpha \sum_l D_l s_l\right) . \tag{17}$$

This fitness has the form of a standard Ising Hamiltonian with inter-spin couplings and local fields, albeit with the convention difference in overall sign between fitness and energy.

3.1.2 First-order small-coupling expansion

We next consider the general Ising Hamiltonian with inter-spin couplings and local fields

$$H(\vec{s}) = -\frac{1}{2}\epsilon \sum_{i\neq j} J_{ij} s_i s_j - \sum_i h_i s_i\,, \tag{18}$$

where ϵ is a constant to be employed in a small-coupling expansion. With this Hamiltonian, taking thermal energy $k_{\mathrm{B}}T = 1$, the equilibrium probability of finding a particular sequence $\vec{s}$ is

$$P(\vec{s}) = \frac{1}{Z} e^{-H(\vec{s})}, \tag{19}$$

where $Z = \sum_{\vec{s}} e^{-H(\vec{s})}$.

Introducing $F = -\log Z$, we have

$$\frac{\partial F}{\partial h_i} = -\langle s_i \rangle = -m_i \, ,$$
$$\frac{\partial^2 F}{\partial h_i \partial h_j} = -\frac{\partial m_i}{\partial h_j} = \langle s_i \rangle \langle s_j \rangle - \langle s_i s_j \rangle = -C'_{ij} \, , \tag{20}$$

where, following the Ising terminology, m_i denotes the average magnetization at site i, while C' denotes the covariance matrix in the Ising convention. Note that, using the identity $m_i = 2P_i - 1$, where P_i denotes the probability that $s_i = 1$, we obtain

$$C'_{ij} = \langle s_i s_j \rangle - \langle s_i \rangle \langle s_j \rangle = 4\,(P_{ij} - P_i P_j) = 4\, C_{ij} \, , \tag{21}$$

where P_{ij} is the probability that $s_i = s_j = 1$, and C denotes the covariance matrix in the Potts convention, which is used in the main text because it allows straightforward generalization to the case where more than two states are possible at each site.

Performing a Legendre transform, we introduce $G = F + \sum_i m_i h_i$, yielding

$$\frac{\partial G}{\partial m_i} = h_i \, , \tag{22}$$
$$\frac{\partial^2 G}{\partial m_i \partial m_j} = \frac{\partial h_i}{\partial m_j} = {C'_{ij}}^{-1} \, . \tag{23}$$

We now perform a small-coupling expansion and express G to first order in ϵ (see Eq. 18): $G(\epsilon) \approx G(0) + \epsilon G'(0)$. Since sites are independent for $\epsilon = 0$, it is straightforward to express $G(0)$ and $G'(0)$ as a function of the one-body expectations, represented by m_i, and of the couplings. We obtain

$$G(0) = \sum_i \frac{m_i + 1}{2} \log\left(\frac{m_i + 1}{2}\right) + \frac{1 - m_i}{2} \log\left(\frac{1 - m_i}{2}\right) \, , \tag{24}$$

and

$$G'(0) = \frac{\partial G}{\partial \epsilon}(0) = -\frac{1}{2} \sum_{i \neq j} J_{ij} m_i m_j \, . \tag{25}$$

Using these expressions, and taking $\epsilon = 1$ in the expansion, we obtain the following approximation for G:

$$G \approx \sum_i \frac{m_i + 1}{2} \log\left(\frac{m_i + 1}{2}\right) + \frac{1 - m_i}{2} \log\left(\frac{1 - m_i}{2}\right) - \frac{1}{2} \sum_{i \neq j} J_{ij} m_i m_j \, . \tag{26}$$

Using Eqs. 22 and 23, we obtain the elements of the inverse covariance matrix from Eq. 26:

$${C'_{kl}}^{-1} = -J_{kl} \, , \ \forall l \neq k \, ,$$
$${C'_{ll}}^{-1} = \frac{1}{2}\left(\frac{1}{1 + m_l} + \frac{1}{1 - m_l}\right) = \frac{1}{4}\left(\frac{1}{P_l} + \frac{1}{1 - P_l}\right) \, , \tag{27}$$

where P_l denotes the probability that $s_l = 1$.

Note that Eq. 26 is a first-order small-coupling (or mean-field) approximation. The expansion can be extended to higher order, and the second-order expansion is known as the Thouless, Anderson, and Palmer (TAP) free energy [3, 6].

3.1.3 Application to our sector model

Comparing Eqs. 17 and 18 (with $\epsilon = 1$) allows us to identify the couplings in our sector model as

$$J_{kl} = -\kappa\, D_k D_l = -\kappa\, \Delta_k \Delta_l / 4 \, , \ \forall k \neq l \, . \tag{28}$$

Note that this expression is in the Ising gauge (also known as the zero-sum gauge). Recall also that the link to the Potts convention is made through $C' = 4\,C$ (Eq. 21), which implies ${C'}^{-1} = C^{-1}/4$. Finally, recall that fitness and energy have opposite signs.

Hence, in the Potts convention, Eq. 27 yields for our sector model:

$$C_{kl}^{-1} = \kappa \Delta_k \Delta_l \, , \ \forall l \neq k \, ,$$
$$C_{ll}^{-1} = \frac{1}{P_l} + \frac{1}{1 - P_l} \, . \tag{29}$$

This corresponds to Eq. 9 in the main text.

3.2 Sequences with q possible states at each site

3.2.1 From a sector model to a Potts model for sequences

Motivated by the fact that a real protein sequence has 21 possible states at each site (20 different amino acids plus gap), we now generalize the above result to the case where q states are possible at each site. We denote these states by α with $\alpha \in \{1, .., q\}$. Our sector model can then be mapped to a q-state Potts model. The length-L vector $\vec{\Delta}$ of single-site mutational effects introduced in the two-state case in the main text is replaced by a $(q-1) \times L$ matrix of mutational effects, each being denoted by $\Delta_l(\alpha_l)$. These mutational effects can be measured with respect to a reference sequence $\vec{\alpha}^0$ satisfying $\Delta_l(\alpha_l^0) = 0$, $\forall l \in \{1, \ldots, L\}$: at each site l, the state present in the reference sequence $\vec{\alpha}^0$ serves as the reference with respect to which the mutational effects at that site are measured. For the sake of simplicity, we will take state q as reference state at all sites. This does not lead to any loss of generality, since it is possible to reorder the states for each l.

The generalization of the fitness function Eq. 6 of the main text (Eq. 14) to our q-state model can be written as

$$w(\vec{\alpha}) = -\frac{\kappa}{2}\left(T(\vec{\alpha}) - T^*\right)^2 = -\frac{\kappa}{2}\left(\sum_{l=1}^{L} \Delta_l(\alpha_l) - T^*\right)^2 , \tag{30}$$

(see Eq. 3 in the main text). Expanding this expression, discarding a constant term, and using the fact that there can only be one state at each site, we find that the fitness of sequences can be expressed as

$$w(\vec{\alpha}) = -\frac{\kappa}{2}\sum_{l \neq k} \Delta_l(\alpha_l)\Delta_k(\alpha_k) - \frac{\kappa}{2}\sum_{l=1}^{L} \Delta_l(\alpha_l)\left(\Delta_l(\alpha_l) - 2\,T^*\right) . \tag{31}$$

This is a particular case of the more general Potts Hamiltonian

$$H(\vec{\alpha}) = -\frac{1}{2}\sum_{l \neq k} e_{lk}(\alpha_l, \alpha_k) - \sum_{l=1}^{L} h_l(\alpha_l) , \tag{32}$$

which is the one usually considered in Direct Coupling Analysis (DCA) [1,2].

In order to identify Eq. 31 and Eq. 32, one must deal with the degeneracies present in Eq. 32, where the number of independent parameters is $L(q-1) + L(L-1)(q-1)^2/2$ [7]. To lift this degeneracy, we choose the gauge usually taken in mean-field DCA [2]: $e_{lk}(\alpha_l, q) = e_{lk}(q, \alpha_k) = h_l(q) = 0$ for all l, k, α_l, α_k. This choice is consistent with taking state q as the reference state for mutational effects (see above), and we will refer to it as the reference-sequence gauge. This gauge choice enables us to identify the couplings between Eq. 31 and Eq. 32:

$$e_{lk}(\alpha_l, \alpha_k) = -\kappa\Delta_l(\alpha_l)\Delta_k(\alpha_k) , \tag{33}$$

for all $l \neq k$, and all α_l, α_k, with $\Delta_l(q) = 0$ for all l (recalling that fitness and energy have opposite signs).

3.2.2 First-order small-coupling expansion

The derivation of the first-order mean-field or small-coupling approximation for q-state models is very similar to the Ising case presented above. Hence, we will simply review the main results (see Ref. [2]).

We start with the Hamiltonian

$$H(\vec{\alpha}) = -\frac{\epsilon}{2}\sum_{l \neq k} e_{lk}(\alpha_l, \alpha_k) - \sum_{l=1}^{L} h_l(\alpha_l) , \tag{34}$$

where ϵ has been introduced to perform the small-coupling expansion. Eq. 34 coincides with Eq. 32 for $\epsilon = 1$. Considering $F = -\log(Z)$ with $Z = \sum_{\vec{\alpha}} e^{-H(\vec{\alpha})}$, where $H(\vec{\alpha})$ is the Potts Hamiltonian in Eq. 34, we have for all k and all $\alpha_k < q$:

$$\frac{\partial F}{\partial h_k(\alpha_k)} = -P_k(\alpha_k) , \tag{35}$$

where $P_k(\alpha_k)$ is the one-body probability. Similarly, we have for all k, l and all $\alpha_k < q$ and $\alpha_l < q$:

$$\frac{\partial^2 F}{\partial h_l(\alpha_l)\partial h_k(\alpha_k)} = -\frac{\partial P_k(\alpha_k)}{\partial h_l(\alpha_l)} = -C_{kl}(\alpha_k, \alpha_l) , \tag{36}$$

where we have introduced the covariance $C_{kl}(\alpha_k, \alpha_l) = P_{kl}(\alpha_k, \alpha_l) - P_k(\alpha_k)P_l(\alpha_l)$.

We perform a Legendre transform and introduce $G = F - \sum_i \sum_{\alpha_i} h_i(\alpha_i)P_i(\alpha_i)$, yielding

$$\frac{\partial G}{\partial P_k(\alpha_k)} = h_k(\alpha_k)\,, \tag{37}$$

$$\frac{\partial^2 G}{\partial P_l(\alpha_l)\partial P_k(\alpha_k)} = \frac{\partial h_l(\alpha_l)}{\partial P_k(\alpha_k)} = C^{-1}_{kl}(\alpha_k, \alpha_l)\,, \tag{38}$$

for all k, l and all $\alpha_k < q$ and $\alpha_l < q$. Note that, in the latter equation, $C^{-1}_{kl}(\alpha, \beta)$ is shorthand for A^{-1}_{ij}, where A is the $(q-1)L \times (q-1)L$ covariance matrix where terms involving the reference state q have been excluded: $A_{ij} = C_{kl}(\alpha, \beta)$, where $i = (q-1)(k-1) + \alpha$ and $j = (q-1)(l-1) + \beta$, with $\alpha \in \{1, \ldots, q-1\}$ and $\beta \in \{1, \ldots, q-1\}$ [8].

We next perform a first-order expansion of G in ϵ, and take $\epsilon = 1$, yielding:

$$G \approx \sum_l \sum_{\alpha_l} P_l(\alpha_l) \log\left(P_l(\alpha_l)\right) - \frac{1}{2} \sum_{l \neq k} \sum_{\alpha_l, \alpha_k} e_{lk}(\alpha_l, \alpha_k) P_l(\alpha_l) P_k(\alpha_k)\,. \tag{39}$$

Applying Eqs. 37, 38 to Eq. 39, and using $P_l(q) = 1 - \sum_{\alpha_l < q} P_l(\alpha_l)$ gives

$$\begin{aligned} C^{-1}_{kl}(\alpha_k, \alpha_l) &= -e_{kl}(\alpha_k, \alpha_l),\ \ \forall\, l \neq k\,, \\ C^{-1}_{ll}(\alpha_k, \alpha_l) &= \frac{1}{P_k(q)} + \frac{\delta_{\alpha_k \alpha_l}}{P_k(\alpha_k)}\,. \end{aligned} \tag{40}$$

This result is the standard one found in DCA [2].

3.2.3 Application to our sector model

Combining Eqs. 33 and 40, we obtain for our sector model:

$$\begin{aligned} C^{-1}_{kl}(\alpha_k, \alpha_l) &= \kappa \Delta_k(\alpha_k) \Delta_l(\alpha_l),\ \ \forall\, l \neq k\,, \\ C^{-1}_{ll}(\alpha_k, \alpha_l) &= \frac{1}{P_k(q)} + \frac{\delta_{\alpha_k \alpha_l}}{P_k(\alpha_k)}\,. \end{aligned} \tag{41}$$

For $q = 2$, Eq. 41 reduces to Eq. 27 (Eq. 9 in the main text), using $1 - P_l = P_l(q)$.

3.2.4 Selection on multiple traits

So far, we have mainly discussed the case where there selection on only one trait (yielding one sector). However, real proteins face various selection pressures. The generalization of the fitness in Eq. 30 to N simultaneous selection on different traits reads

$$w(\vec{S}) = -\sum_{i=1}^{N} \frac{\kappa_i}{2} \left(T_i - T_i^*\right)^2 = -\sum_{i=1}^{N} \frac{\kappa_i}{2} \left(\sum_{l=1}^{L} \Delta_{i,l}(\alpha_l) - T_i^* \right)^2\,, \tag{42}$$

which corresponds to Eq. 11 in the main text. We choose the reference-state gauge, assuming again for simplicity that the reference state is q at each site. The identification to the general Potts Hamiltonian Eq. 32 (recalling that fitnesses and energies have opposite signs) then yields

$$e_{lk}(\alpha_l, \alpha_k) = -\sum_{i=1}^{N} \kappa_i \Delta_{i,l}(\alpha_l) \Delta_{i,k}(\alpha_k)\,, \tag{43}$$

which generalizes Eq. 33 to the multiple selection case. Using the small-coupling expansion result in Eq. 40, we obtain the following approximation for the inverse covariance matrix:

$$\begin{aligned} C^{-1}_{kl}(\alpha_k, \alpha_l) &= \sum_{i=1}^{N} \kappa_i \Delta_{i,k}(\alpha_k) \Delta_{i,l}(\alpha_l),\ \ \forall\, l \neq k\,, \\ C^{-1}_{ll}(\alpha_k, \alpha_l) &= \frac{1}{P_k(q)} + \frac{\delta_{\alpha_k \alpha_l}}{P_k(\alpha_k)}\,. \end{aligned} \tag{44}$$

This generalizes Eq. 41 to the case of simultaneous selection on multiple traits.

4 Robustness of functional sectors and of ICOD

In the main text, we introduced the Inverse Covariance Off-Diagonal (ICOD) method to identify protein sectors from sequence data. The ICOD method exploits the approximate expression derived above for the inverse covariance matrix (Eq. 41); in particular, ICOD makes use of the fact that the off-diagonal elements of C^{-1} are simply related to the elements of the mutational effect vector $\vec{\Delta}$. In this section, we first describe our comparison of ICOD to SCA for single selection, and detail our test of ICOD for double selection, using synthetic binary sequences. Next, we confirm the robustness of the ICOD method to different forms of selection and then show how ICOD can be extended to sequences with more than two states per site, and finally demonstrate its robustness to gauge choice and pseudocounts.

4.1 Robustness of ICOD to selection bias, selection strength, and multiple selections

To quantify the performance of ICOD and to compare to SCA over a range of selection biases we focused on binary sequences. To obtain the average curve for single selections in Fig. 3(a) of the main text, we first generated 100 distinct synthetic $\vec{\Delta}$s, one for each sector size from $n = 1$ to 100, where sector sites are defined as those with large mutational effects. To this end, the mutational effects of the sector sites and the non-sector sites were sampled, respectively, from zero-mean Gaussian distributions with standard deviations 20 and 1. For each sector size and each selection bias we generated a sequence ensemble of 50,000 random sequences and weighted each sequence according to the distribution

$$P(\vec{S}) = \frac{\exp(w(\vec{S}))}{\sum_{\vec{S}} \exp(w(\vec{S}))}\,, \tag{45}$$

where $w(\vec{S})$ is the fitness of sequence $\vec{S}$, given by the single selection formula Eq. 6 in the main text. In general, we wish to employ a selection window whose width in energy (or any other selected variable) scales with the overall width of the unselected distribution. Hence, as mentioned in the main text, we perform all selections with a strength

$$\kappa = \frac{10}{\sum_l \Delta_l^2}\,. \tag{46}$$

Then, for each method (ICOD or SCA), performance as measured by Recovery of $\vec{\Delta}$ by the first eigenvector was averaged over the 100 different sector sizes.

As an aside, Fig. 3 demonstrates that the performance of ICOD and SCA is robust to varying selection strength κ, as long as $\kappa \sum_l \Delta_l^2 \gg 1$. (A small value of $\kappa \sum_l \Delta_l^2$ implies weak selection, where most random sequences pass selection and the resulting ensemble does not significantly reflect the constraint.)

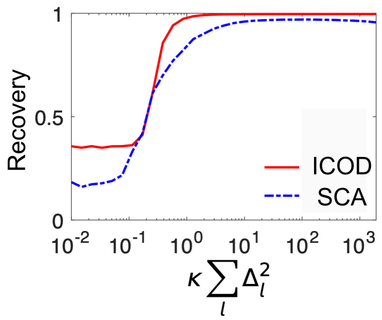

Fig 3. Impact of selection strength κ on the performance of ICOD and SCA on synthetic data. Results obtained on binary synthetic sequences with $L = 100$, selected using a synthetic $\vec{\Delta}$ where the first 20 and the other 80 mutational effects are, respectively, sampled from Gaussian distributions with variances of 20 and 1. Selection is performed on ensembles of 50,000 random sequences, and each data point is obtained by averaging over 100 realizations. The relative bias is $\gamma = 0.5$.

Similarly, to obtain the average curve for double selection in Fig. 3(b) of the main text, we generated 100 distinct pairs of $\vec{\Delta}_1$s and $\vec{\Delta}_2$s, one pair for each sector size from $n = 1$ to 100. Specifically, the sector for $\vec{\Delta}_1$ consisted of the first n sites, while the sector for $\vec{\Delta}_2$ corresponded to the last n sites, so that the two sectors overlap for $n > 50$. As for the single selections, the mutational effects of the sector sites and the non-sector sites were sampled, respectively, from Gaussian distributions with standard deviations 20 and 1. As an example, two synthetic $\vec{\Delta}$s for $n = 20$ are

shown in Fig. 4. Again, for each sector size and each selection bias, we generated an ensemble of 50,000 random sequences and weighted them according to Eq. 45 along with the double selection formula Eq. 42 (i.e. Eq. 11 in the main text). The performance of ICOD as measured by Recovery of $\vec{\Delta}_1$ and $\vec{\Delta}_2$ by the first two eigenvectors was averaged over the 100 different sector sizes. In Fig. 3(b) of the main text we also reported the performance of ICOD for two non-overlapping sectors, each with 20 sites, and for two fully overlapping sectors, each with 100 sites. We followed a protocol similar to that described above, but in each of these cases, we averaged Recovery over 100 realizations using distinct pairs of $\vec{\Delta}_1$ and $\vec{\Delta}_2$.

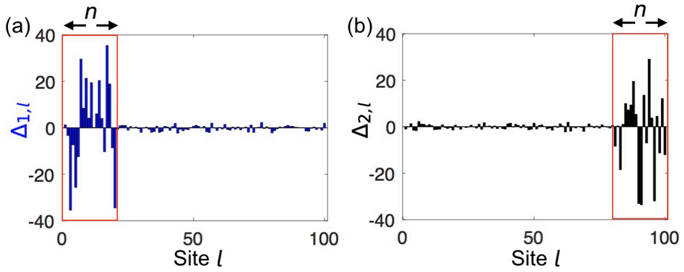

Fig 4. Example of two synthetic $\vec{\Delta}$s generated for the double selection in Fig. 3(b) of the main text. (a) Generation of $\vec{\Delta}_1$, where the mutational effects for the first 20 sites and for the last 80 sites are sampled, respectively, from zero-mean Gaussian distributions with a standard deviation of 20 and 1. (b) Generation of $\vec{\Delta}_2$, where the mutational effects for the last 20 sites and for the first 80 sites are sampled, respectively, from zero-mean Gaussian distributions with a standard deviation of 20 and 1.

Unless otherwise stated, data for other plots were generated in the same way, i.e. using 50,000 random sequences, sequence length $L = 100$, selection strength κ in Eq. 46, and standard deviation 20/1 of Δ_l in the sector/non-sector sites.

Note that to improve Recovery in the case of double selection, we applied Independent Component Analysis (ICA) [9–11] to the first two eigenvectors in order to disentangle the contributions coming from the two constraints. In general, we expect that the first N eigenvectors of the ICOD matrix $\tilde{C}^{-1}$ will report N constraints. However, each of these N eigenvectors is likely to include a mixture of contributions from different constraints. Applying ICA to the first N eigenvectors to recover the individual constraints amounts to assuming that all the constraints are statistically independent. As an example, in Fig. 5, we consider the case of two selections targeting a different set of sites and with different selection windows (one biased, one non-biased). In this case, ICOD plus ICA yields excellent Recovery (Fig. 5). Without ICA, the results are noticeably worse (Fig. 6). Moreover, Fig. 3(b) of the main text demonstrates that ICOD plus ICA can achieve a high Recovery for a broad range of overlaps between two sectors.

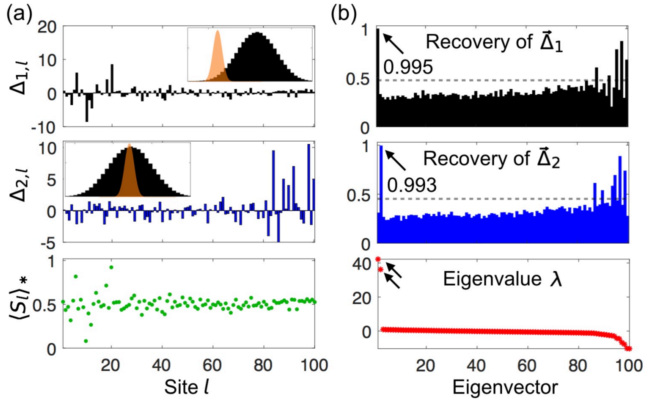

Fig 5. ICOD method for simultaneous selection on two traits. (a) Upper panels: Components at each site l of two synthetically generated mutational-effect vectors, with insets showing biased selection around T_1^* for $\vec{\Delta}_1$ and neutral selection around T_2^* for $\vec{\Delta}_2$. Lower panel: average mutant fraction $\langle S_l \rangle_*$ at site l after selection on both traits. (b) Performance of ICOD method. Recovery of $\vec{\Delta}_1$ and $\vec{\Delta}_2$ for all eigenvectors (upper) and corresponding eigenvalues (lower). The gray dashed line indicates the random expectation of Recovery (Eq. 11).

In Fig. 3(b) of the main text, one observes a slight decrease of performance of ICOD plus ICA for double selection

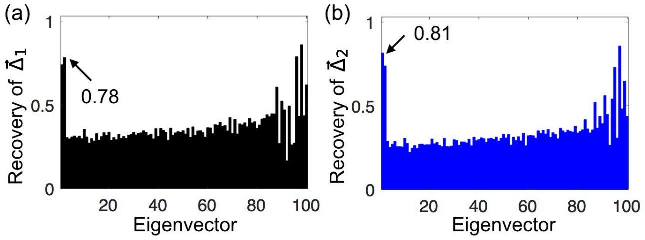

Fig 6. Performance of ICOD for the two-sector case in Fig. 5, without applying ICA.

with overlapping sectors. Does this arise from increasing sector size or from increasing overlap? As expected from Eqs. 8 and 9 of the main text, Fig. 7(a) shows that Recovery does not fall off with increased sector size. Thus, we tested whether larger sector overlaps could reduce Recovery. Fig. 7(b) shows that this is indeed the case for sequence ensembles subject to two selections each with a fixed sector size of 20, but with different numbers of overlapping sites. However, the reduction of Recovery is quite modest, as even for 100% overlap, Recovery remains above 0.9. It is interesting to note that, independent of sector size and overlap, Recovery decreases faster for double selection than for single selection at large relative biases (see Fig. 3 in the main text and Fig. 7).

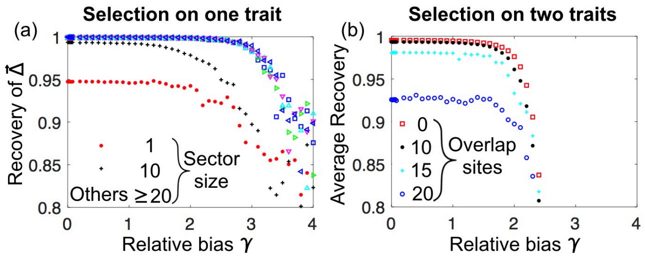

Fig 7. Performance of ICOD for different sector sizes and sector overlaps. (a) Selection on a single trait with varying sector size. Recovery is shown as a function of relative selection bias $\gamma \equiv (T^* - \langle T \rangle)/\sqrt{\langle (T - \langle T \rangle)^2 \rangle}$ for sectors of size 1, 10, 20, 40, 60, 80, and 100 out of 100 sequence sites (cf. Fig. 3(a) of the main text). Recovery is almost perfect for sectors of size larger than 10, but is substantially lower for sector size 1, which violates the criteria $\Delta_l \ll \sqrt{\sum_{l'} \Delta_{l'}^2}$. (b) Simultaneous selection on two traits with different degrees of sector overlap. For each selection, the sector size is 20 out of 100 sequence sites, and the overlap varies from 0 to 20 sites. The average Recovery for $\vec{\Delta}_1$ and $\vec{\Delta}_2$ is shown as a function of relative selection bias. The data in (b) is averaged over 20 realizations of $\vec{\Delta}$s.

4.2 Robustness of functional sectors to different forms of selection

To assess the robustness of physical sectors to forms of selection other than the simple Gaussian selection window of Eqs. 2-3 of the main text, we generated ensembles of 50,000 random binary sequences as above, and used synthetically generated mutational effects, with 20 sector sites out of $L = 100$ total sites. As before, the mutational effects of the sector sites and the non-sector sites were sampled, respectively, from zero-mean Gaussian distributions with standard deviations 20 and 1.

We first addressed selection for sequences with an additive trait T above a threshold T_t. We thus considered the selected ensembles of sequences such that the value of the trait $T(\vec{S}) = \vec{S} \cdot \vec{\Delta}$ is larger than a threshold T_t, and we varied this threshold. Fig. 4 in the main text demonstrates that the corresponding sectors are identified by ICOD just as well as those resulting from our initial Gaussian selection window.

We also successfully applied ICOD to various other forms of selection. In Fig. 8, we used the quartic fitness function:

$$w(\vec{S}) = -\frac{\kappa_1}{4}\left(\sum_{l=1}^{L} \Delta_l S_l - T^*\right)^4 , \quad (47)$$

with $\kappa_1 = (10/\sum_l \Delta_l^2)^2$, instead of our initial quadratic fitness function (see Eq. 14, and Eq. 3 in the main text) and we weighted sequences using the Boltzmann distribution in Eq. 2 of the main text. Finally, in Fig. 9, we considered the selected ensembles of sequences such that the value of the trait $T(\vec{S}) = \vec{S} \cdot \vec{\Delta}$ is between $T^* - \eta/2$ and $T^* + \eta/2$, where η is the width of the selection window. In Fig. 9, we used $\eta = 0.6\sqrt{\sum_l \Delta_l^2}$.

These results confirm the robustness of our approach to different plausible forms of selection.

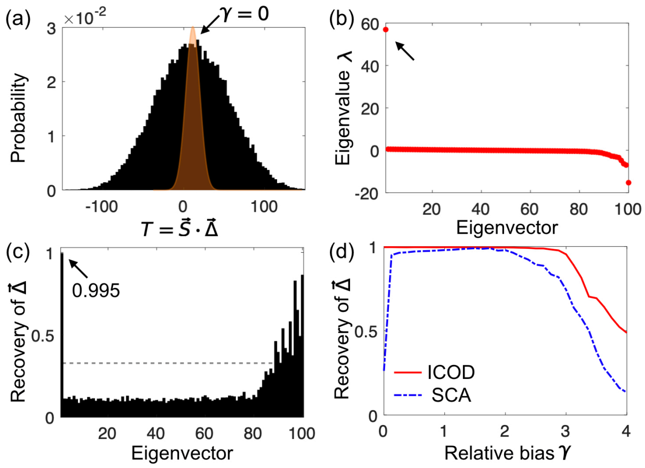

Fig 8. Identification of sectors that result from quartic selection. (a) Histogram of the additive trait $T(\vec{S}) = \vec{S} \cdot \vec{\Delta}$ for randomly sampled sequences where 0 and 1 are equally likely at each site. Sequence length is $L = 100$, mutational effects are synthetically generated with 20 sector sites. Sequences are selectively weighted using a quartic window (orange) around T^*. Selection is shown for $T^* = \langle T \rangle$, or equivalently $\gamma = 0$, in terms of the relative selection bias $\gamma \equiv (T^* - \langle T \rangle)/\sqrt{\langle (T - \langle T \rangle)^2 \rangle}$. (b) Eigenvalues of the ICOD-modified inverse covariance matrix $\tilde{C}^{-1}$ (Eq. 10 of the main text) of the selected sequences for $\gamma = 0$. (c) Recovery of $\vec{\Delta}$ for all eigenvectors of $\tilde{C}^{-1}$ for $\gamma = 0$. Gray dashed line: random expectation of Recovery. (d) Recovery of $\vec{\Delta}$ for ICOD and for SCA as functions of the relative selection bias γ. The data in (d) is averaged over 100 realizations of $\vec{\Delta}$.

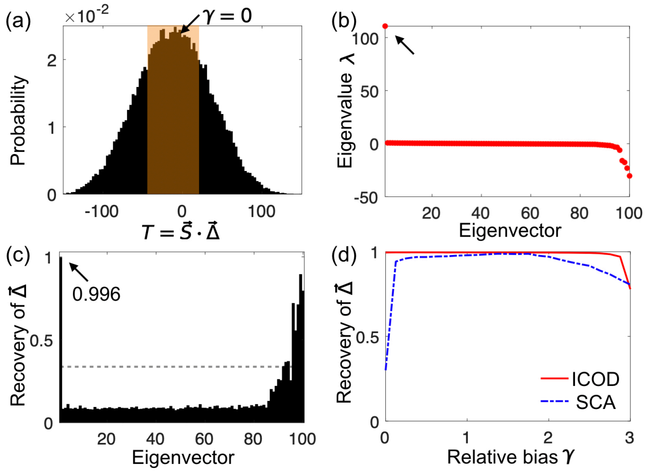

Fig 9. Identification of sectors that result from rectangular-window selection. (a) Histogram of the additive trait $T(\vec{S}) = \vec{S} \cdot \vec{\Delta}$ for randomly sampled sequences where 0 and 1 are equally likely at each site. Sequence length is $L = 100$, mutational effects are synthetically generated with 20 sector sites. Sequences are selected if they have a trait value $T^* - \eta/2 < T(\vec{S}) < T^* + \eta/2$ (orange shaded region). Selection is shown for $T^* = \langle T \rangle$, or equivalently $\gamma = 0$, in terms of the relative selection bias $\gamma \equiv (T^* - \langle T \rangle)/\sqrt{\langle (T - \langle T \rangle)^2 \rangle}$. (b) Eigenvalues of the ICOD-modified inverse covariance matrix $\tilde{C}^{-1}$ (Eq. 10 of the main text) of the selected sequences for $\gamma = 0$. (c) Recovery of $\vec{\Delta}$ for all eigenvectors of $\tilde{C}^{-1}$ for $\gamma = 0$. Gray dashed line: random expectation of Recovery. (d) Recovery of $\vec{\Delta}$ for ICOD and for SCA as functions of the relative selection threshold γ. The data in (d) is averaged over 100 realizations of $\vec{\Delta}$.

4.3 Multiple states per site and alternative gauge choice

In Section 3.2 above, we described how to generalize from binary sequences to sequences with q possible states at each site. Correspondingly, we now generalize the ICOD method to higher values of q. Since we are interested in extracting the single-site mutational effects $\Delta_l(\alpha_l)$ with respect to a reference state at each site, we can simply set to zero the diagonal blocks of C^{-1} in Eq. 44, yielding the modified inverse covariance matrix

$$\tilde{C}^{-1}_{kl}(\alpha_k, \alpha_l) = (1 - \delta_{kl}) \sum_{i=1}^{N} \kappa_i \Delta_{i,k}(\alpha_k)\Delta_{i,l}(\alpha_l)\,, \tag{48}$$

for the case of multiple selections, or more simply for a single selection

$$\tilde{C}^{-1}_{kl}(\alpha_k, \alpha_l) = (1 - \delta_{lk})\, \kappa \Delta_k(\alpha_k)\Delta_l(\alpha_l). \tag{49}$$

This equation generalizes Eq. 10 of the main text, which was obtained for $q = 2$. As in that case, the first eigenvector of $\tilde{C}^{-1}$ (associated with the largest eigenvalue) should accurately report the single-site mutational effects $\Delta_k(\alpha_k)$. Indeed, Fig. 10 in the main text shows that this generalized version of ICOD performs very well on synthetic data generated for the case $q = 21$ relevant to real protein sequences. Note that in the reference-sequence gauge, Recovery generalizes naturally to the q-state model as

$$\text{Recovery} = \frac{\sum_{l,\alpha_l} |\nu_l(\alpha_l)\Delta_l(\alpha_l)|}{\sqrt{\sum_{l,\alpha_l} \nu_l(\alpha_l)^2}\sqrt{\sum_{l,\alpha_l} \Delta_l(\alpha_l)^2}}, \tag{50}$$

where the sums over states α_l do not include the reference state at each site.

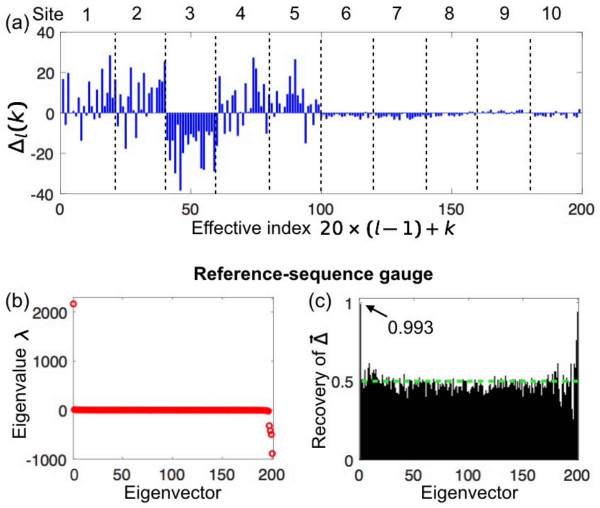

Fig 10. Performance of ICOD on synthetic sequence data with $q = 21$ possible states at each site. (a) Mutational effects $\Delta_l(k)$ with respect to a reference sequence, chosen to be state 21 at every site. The mutational effect at $q = 21$ is not shown. Note that while mutational effects are initially generated from a Gaussian distribution, *relative* mutational effects (calculated with respect to the reference sequence) can have a systematic bias at each site l. (b) Eigenvalues of the ICOD-modified inverse covariance matrix $\tilde{C}^{-1}$ defined in Eq. 49. (c) Recovery of $\vec{\Delta}$ (see Eq. 50). The green dashed line indicates the random expectation of Recovery (Eq. 11).

While the reference-sequence gauge is convenient and allows a clear interpretation of the mutational effects, other gauge choices are possible. For instance, in the DCA literature, the zero-sum (or Ising) gauge is often employed [8,12]. In this gauge, the couplings satisfy

$$\sum_{\alpha} e_{ij}(\alpha, \beta) = \sum_{\beta} e_{ij}(\alpha, \beta) = 0\,, \tag{51}$$

Qualitatively, the gauge degree of freedom means that contributions to the Hamiltonian in Eq. 32 can be shifted between the fields and the couplings [13]. In DCA, the focus is on identifying the dominant two-body interactions,

so one does not want the couplings to include contributions that can be accounted for by the one-body fields [7]. The zero-sum gauge satisfies this condition because it minimizes the Frobenius norms of the couplings

$$\|e_{ij}\| = \sqrt{\sum_{\alpha,\beta=1}^{q} \left[e_{ij}(\alpha,\beta)\right]^2}\,. \tag{52}$$

Hence, the zero-sum gauge attributes the smallest possible fraction of the energy in Eq. 32 to the couplings, and the largest possible fraction to the fields [8,13]. In order to transform to the zero-sum gauge defined in Eq. 51, each coupling $e_{ij}(\alpha,\beta)$ is replaced by

$$\tilde{e}_{ij}(\alpha,\beta) = e_{ij}(\alpha,\beta) - \langle e_{ij}(\zeta,\beta)\rangle_\zeta - \langle e_{ij}(\alpha,\eta)\rangle_\eta + \langle e_{ij}(\zeta,\eta)\rangle_{\zeta,\eta}\,, \tag{53}$$

where $\langle . \rangle_\zeta$ denotes an average over $\zeta \in \{1, ..., q\}$ [8].

Shifting from the reference-sequence gauge where one state (in our derivations, state q) is taken as a reference at each site to the zero-sum gauge requires the replacement

$$\tilde{\Delta}_l(\alpha) = \Delta_l(\alpha) - \frac{1}{q}\sum_{\beta=1}^{q} \Delta_l(\beta), \tag{54}$$

The new reference-state-free mutational effects satisfy $\sum_{\beta=1}^{q} \tilde{\Delta}_l(\beta) = 0$, and the associated couplings $\tilde{e}_{lk}(\alpha_l, \alpha_k) = -\kappa\tilde{\Delta}_l(\alpha_l)\tilde{\Delta}_k(\alpha_k)$ (see Eq. 33) are related to the initial ones $e_{lk}(\alpha_l, \alpha_k)$ through Eq. 53.

Importantly, these reference-state-free mutational effects can be used to assess the overall importance of mutations at each particular site in the sequence. To this end, let us introduce the Frobenius norm of the reference-state-free mutational effects:

$$||\Delta_l|| = \sqrt{\sum_{\beta=1}^{q} \left(\tilde{\Delta}_l(\beta)\right)^2}\,. \tag{55}$$

This quantity, which we refer to as the "site significance", measures the overall importance of mutational effects at site l. In order to assess site significances from an ensemble of sequences, without investigating the impact of each particular mutation at each site, one can apply the zero-sum gauge to the ICOD-modified inverse covariance matrix (see Eq. 49), and compute the Frobenius norm of each 20×20 block associated to each pair of sites (i, j) according to Eq. 52. The first eigenvector of this compressed $L \times L$ matrix accurately reports the mutational significance of each site, as illustrated in Fig. 11. Specifically, it yields a high Recovery of site significances as defined in Eq. 55 (see Fig. 11(c)), and it successfully predicts the most important sites, i.e. the sector sites, in our synthetic data (see Fig. 11(d)).

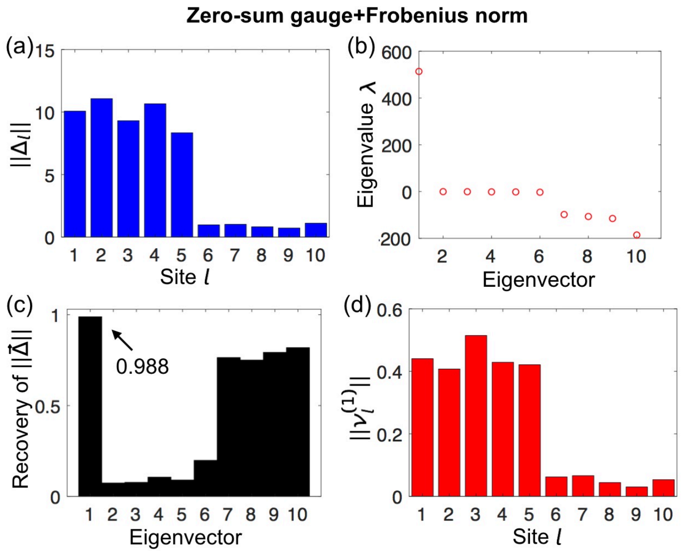

Fig 11. Assessing site significance for synthetic sequence data. The same synthetic data as in Fig. 10 (with $q = 21$ possible states at each site) is used. (a) Significance $||\Delta_l||$ of each site l, computed directly by applying Eqs. 54 and 55 to the mutational effects $\Delta_l(k)$ shown in Fig. 10(a). (b) Eigenvalues of the compressed $(L \times L)$ ICOD-modified inverse covariance matrix, calculated by applying the zero-sum gauge to the ICOD-modified inverse covariance matrix (see Eq. 49), and by computing the Frobenius norm of each 20×20 block associated to each pair of sites (i, j) according to Eq. 52. (c) Recovery of site significances $||\vec{\Delta}||$ from each eigenvector of the compressed ICOD-modified inverse covariance matrix (see panel (a) and Eq. 50) (d) Estimated site significances computed from the first eigenvector $\vec{\nu}^{(1)}$ of the compressed ICOD-modified inverse covariance matrix.

4.4 Pseudocounts

As pseudocounts are often necessary to regularize real sequence data, and as a high fraction of pseudocounts is generally used in DCA, we consider here whether the ICOD method is robust to the addition of pseudocounts.

Until now, we used only raw empirical frequencies obtained from sequence data. For instance, one-body frequencies were obtained by counting the number of sequences where a given state occured at a given site and dividing by the total number M of sequences in the ensemble. Covariances were computed from the empirical single-site frequencies of occurrence of each state α at each site i, denoted by $f_i^e(\alpha)$, and the empirical two-site frequencies of occurrence of each ordered pair of states (α, β) at each ordered pair of sites (i, j), denoted by $f_{ij}^e(\alpha, \beta)$. Specifically, we obtained the covariance matrix as $C_{ij}(\alpha, \beta) = f_{ij}^e(\alpha, \beta) - f_i^e(\alpha) f_j^e(\beta)$ [13].

To avoid issues arising from limited sample size, such as states that never appear at some sites (which present mathematical difficulties, e.g. a non-invertible covariance matrix [2]), one can introduce pseudocounts via a parameter Λ [1, 2, 13, 14]. The one-site frequencies f_i then become

$$f_i(\alpha) = \frac{\Lambda}{q} + (1 - \Lambda) f_i^e(\alpha)\,, \tag{56}$$

where q is the number of states per site. Similarly, the two-site frequencies f_{ij} become

$$f_{ij}(\alpha, \beta) = \frac{\Lambda}{q^2} + (1 - \Lambda) f_{ij}^e(\alpha, \beta) \text{ if } i \neq j\,, \tag{57}$$

$$f_{ii}(\alpha, \beta) = \frac{\Lambda}{q}\delta_{\alpha\beta} + (1 - \Lambda) f_{ii}^e(\alpha, \beta) = f_i(\alpha)\delta_{\alpha\beta}\,. \tag{58}$$

These pseudocount corrections are uniform (i.e. they have the same weight $1/q$ for all states), and their influence relative to the raw empirical frequencies can be tuned through the parameter Λ. In DCA, a high value of f Λ has been found to improve contact prediction: typically $\Lambda \approx 0.5$ [1, 2, 15]. Note that the correspondence of Λ with the parameter λ in Refs. [1, 2, 14] is obtained by setting $\Lambda = \lambda/(\lambda + M)$.

From these quantities, we define the pseudocount-corrected covariances

$$C'_{ij}(\alpha, \beta) = f_{ij}(\alpha, \beta) - f_i(\alpha) f_j(\beta)\,. \tag{59}$$

We show in Fig. 12 that adding pseudocounts as high as $\Lambda = 0.3$ still allows for accurate extraction of mutational effects (Recovery 0.96) and provides a reliable prediction of sector sites.

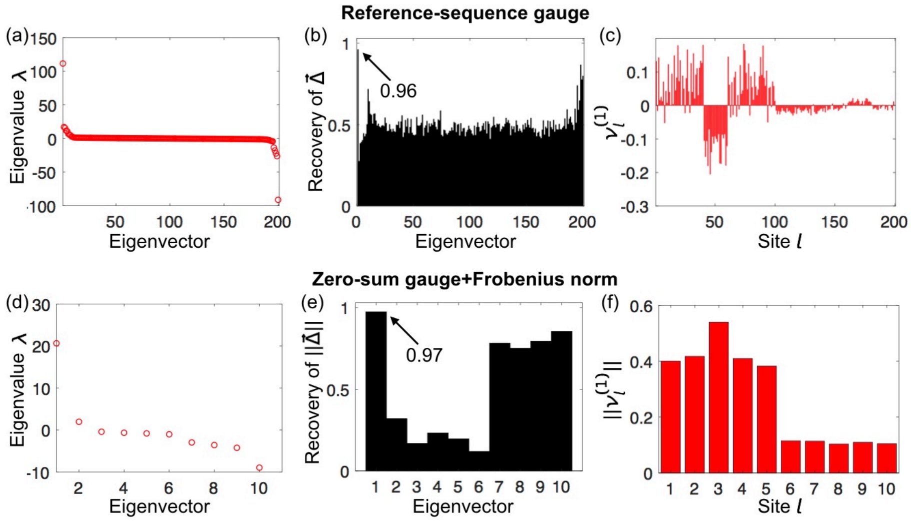

Fig 12. Effect of pseudocounts on ICOD performance on synthetic sequence data with $q = 21$ possible states at each site. The same synthetic data as in Fig. 10 and 11 is used, but here pseudocounts are employed, with weight $\Lambda = 0.3$. (a-c) Similar analysis as in Fig. 10: (a) Eigenvalues of the ICOD-modified inverse covariance matrix. (b) Recovery of $\vec{\Delta}$ from each eigenvector of the ICOD-modified inverse covariance matrix. (c) First eigenvector of the ICOD-modified inverse covariance matrix. (d-f) Similar analysis as in Fig. 11: (d) Eigenvalues of the compressed ICOD-modified inverse covariance matrix. (e) Recovery of site significances $||\vec{\Delta}||$ from each eigenvector of the compressed ICOD-modified inverse covariance matrix. (f) Estimated site significances computed from the first eigenvector of the compressed ICOD-modified inverse covariance matrix.

5 Performance of SCA

5.1 Analytical estimates for $\langle S_l \rangle_*$ and $C_{ll'}$ for a single selection with binary sequences

Protein sectors were first discovered from sequence data using a PCA-based method called Statistical Coupling Analysis (SCA) [11, 16]. Interestingly, in SCA, sectors are found from the eigenvectors associated to the largest eigenvalues, while in ICOD they are found from the (modified) eigenvectors associated to the smallest eigenvalues. This difference stems from the fact that SCA and ICOD do not start from the same matrix. For binary sequences, SCA uses the absolute value of a conservation-weighted covariance matrix, $\tilde{C}^{(\mathrm{SCA})}_{ll'} = |\phi_l C_{ll'} \phi_{l'}|$ (see main text and Ref. [16]). When all amino-acid states are accounted for, SCA compresses each block of the conservation-weighted matrix corresponding to two sites to obtain one positive value, e.g. the Frobenius norm of the block [11]. Conversely, ICOD employs the regular covariance matrix, suppressing the diagonal blocks of its inverse at the last step before diagonalization. To better understand the performance of SCA in recovering the site-dependent mutational effects associated with a selective constraint, it is helpful to have analytical estimates for the average mutant fraction $\langle S_l \rangle_*$ at each site l and the covariance matrix $C_{ll'}$ for an ensemble of binary sequences obtained from a single selection using vector of mutational effects $\vec{\Delta}$. To this end, we provide the following two ansatzes:

$$\langle S_l \rangle_* - \langle S_l \rangle \approx (T^* - \langle T \rangle) \frac{\Delta_l}{\sum_l \Delta_l^2}, \tag{60}$$

$$C_{ll'} \approx \begin{cases} -\frac{\Delta_l \Delta_{l'} \sigma_l^2 \sigma_{l'}^2}{\sum_l \Delta_l^2 \sigma_l^2}, & l \neq l' \\ \sigma_l^2, \quad l = l', \end{cases} \tag{61}$$

where $\sigma_l^2 = \langle S_l^2 \rangle_* - \langle S_l \rangle_*^2 = \langle S_l \rangle_* (1 - \langle S_l \rangle_*)$ represents the variance of S_l. Recall that $S_l \in \{0, 1\}$, where 0 is the reference state and 1 the mutant state, and that $\langle \cdot \rangle_*$ denotes ensemble averages over the selectively weighted subset of sequences, while $\langle \cdot \rangle$ denotes averages over the unselected (unweighted) ensemble.

Although we have not proven these two ansatzes, numerical tests (Fig. 13) have verified these two relations for ensembles generated from a $\vec{\Delta}$ with multiple sites of comparably large mutational effects so as not to be dominated by a single site, i.e., $\Delta_l/\sqrt{\sum_{l'}\Delta_{l'}^2} \ll 1$ for any l. As a counterexample, the $\vec{\Delta}$ from our elastic network model does not satisfy this condition.

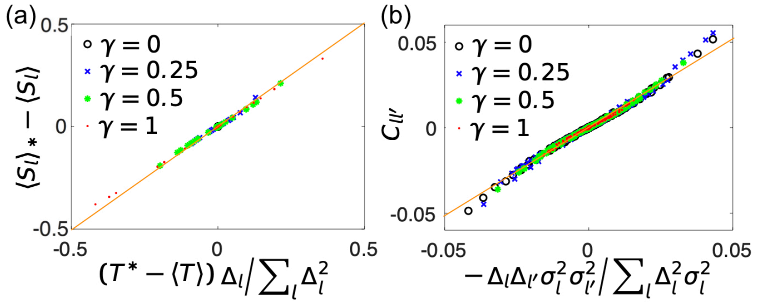

Fig 13. Numerical verification of the ansatzes in Eq. 60 and Eq. 61. We generate a sequence ensemble by considering four values of relative selection bias $\gamma \equiv (T^* - \langle T\rangle)/\sqrt{\langle(T-\langle T\rangle)^2\rangle} = 0, 0.25, 0.5, 1$ and for each case we use a synthetic $\vec{\Delta}$ with a sector size of 20. (a) Numerically computed average bias of the mutant fractions $\langle S_l\rangle_* - \langle S_l\rangle$. Here, $\langle S_l\rangle = 0.5$ for the unselected ensemble. (b) Numerically computed covariances $C_{ll'}$. The results in (a,b) compare well with the analytical predictions (orange lines), provided that $\Delta_l/\sqrt{\sum_l' \Delta_l'^2} \ll 1$ for any l. For each case, 10^6 random sequences were generated to minimize noise from sampling.

5.2 Analysis of the SCA method

Here, we provide a detailed analysis of the SCA method from Refs. [11,16]. Following these references, the reweighting factor is chosen to be

$$\phi_l = \frac{\partial D\left(\langle S_l\rangle_*, \langle S_l\rangle\right)}{\partial\langle S_l\rangle_*}, \tag{62}$$

where, for each site l, $D\left(\langle S_l\rangle_*, \langle S_l\rangle\right)$ is the Kullback-Leibler divergence between the distribution of mutant fractions for the selected sequences and the background distribution:

$$D\left(\langle S_l\rangle_*, \langle S_l\rangle\right) = \langle S_l\rangle_* \log\frac{\langle S_l\rangle_*}{\langle S_l\rangle} + (1-\langle S_l\rangle_*)\log\frac{1-\langle S_l\rangle_*}{1-\langle S_l\rangle}. \tag{63}$$

In our case, the background distribution is obtained from the unselected sequence ensemble, for which $\langle S_l\rangle = 0.5$. Hence, we have

$$\phi_l = \log\left[\frac{\langle S_l\rangle_*\left(1-\langle S_l\rangle\right)}{\langle S_l\rangle\left(1-\langle S_l\rangle_*\right)}\right], \tag{64}$$

as illustrated in Fig. 14(a). In the regime of relatively weak conservation, i.e. when $\langle S_l\rangle$ is not close to 0 or 1, and $|\langle S_l\rangle_* - \langle S_l\rangle| \ll \langle S_l\rangle$, a first-order expansion yields

$$\phi_l \approx \frac{\langle S_l\rangle_* - \langle S_l\rangle}{\langle S_l\rangle(1-\langle S_l\rangle)}, \tag{65}$$

as shown in Fig. 14(b). Employing the ansatz (60) in this regime, we obtain

$$\phi_l \propto (T^* - \langle T\rangle)\Delta_l. \tag{66}$$

This relation is verified in Fig. 14(c) for a sequence ensemble generated with a synthetic Δ_l. Hence, the SCA reweighting factor carries information about Δ_l as long as $T^* \neq \langle T\rangle$. In this regime, information about conservation (namely ϕ_l) should thus be sufficient to recover mutational effects and sectors. This was indeed found to be the case for some real proteins with a single sector [17]. However, when the selection bias, $T^* - \langle T\rangle$, is small, random noise due to finite sampling will typically swamp this relationship.

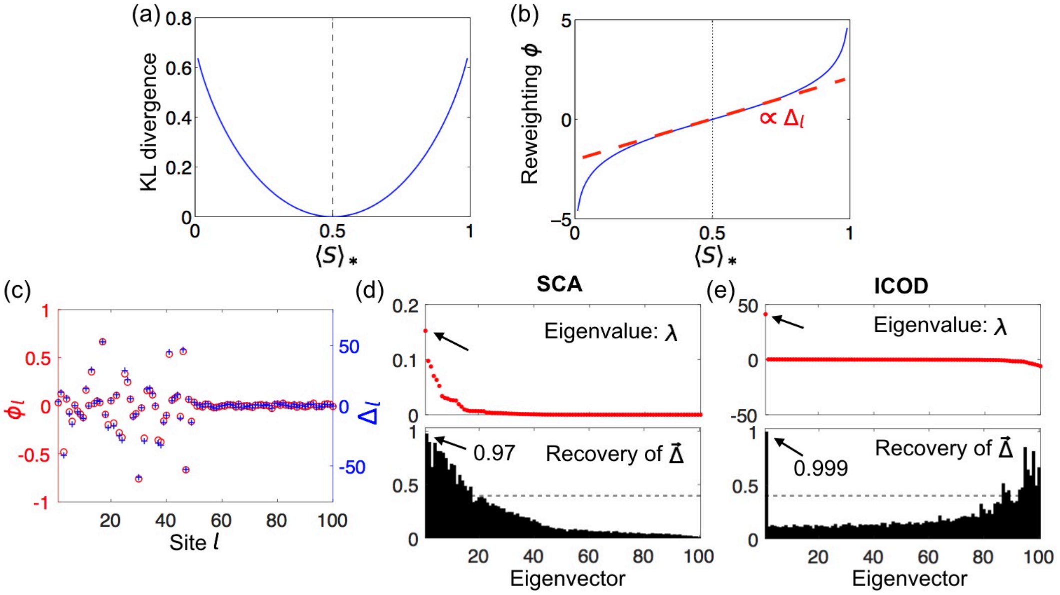

Fig 14. Underpinnings of Recovery of mutational effect vector $\vec{\Delta}$ by SCA. (a) Kullback-Leibler divergence versus mutant fraction $\langle S\rangle_*$ for background mutant fraction $\langle S\rangle = 0.5$. (b) Reweighting factor ϕ as a function of mutant fraction $\langle S\rangle_*$ for background mutant fraction $\langle S\rangle = 0.5$. (c) Reweighting factor ϕ_l and synthetic Δ_l for an ensemble of sequences generated with a single selection at relative selection bias $\gamma = 1$. $\vec{\Delta}$ was generated with the first 50 sites as sector sites, and 50,000 sequences were employed, as in most of our examples using ICOD (see above). (d-e) Performance of SCA and ICOD for this ensemble, respectively. In computing Recovery using SCA, we use the normalized vector $\sqrt{\nu_l^{(j)}}$ to predict $\vec{\Delta}$. The gray dashed lines in (d) and (e) indicate the random expectation of Recovery (Eq. 11).

In Refs. [11,16], the first eigenvectors of the conservation-reweighted SCA covariance matrix, $\tilde{C}^{(\mathrm{SCA})}_{ll'} = |\phi_l C_{ll'} \phi_{l'}|$, were used to find sectors from sequence data. How does the first eigenvector of $\tilde{C}^{(\mathrm{SCA})}$ relate to the mutational effect vector $\vec{\Delta}$? Utilizing both Eq. 61 and Eq. 66, and assuming $T^* \neq \langle T \rangle$, we obtain

$$\tilde{C}^{(\mathrm{SCA})}_{ll'} \propto \begin{cases} \Delta_l^2 \Delta_{l'}^2 \sigma_l^2 \sigma_{l'}^2, & l \neq l' \\ \Delta_l^2 \sigma_l^2, & l = l'. \end{cases} \tag{67}$$

Apart from the diagonal, the matrix is approximately proportional to the tensor product of $\Delta_l^2 \sigma_l^2$ with itself. If we neglect the contribution from the diagonal elements of $\tilde{C}^{(\mathrm{SCA})}$, the first eigenvector $\vec{\nu}^{(1)}$ satisfies

$$\nu_l^{(1)} \propto \Delta_l^2 \sigma_l^2. \tag{68}$$

Eq. 68 explains why $\sqrt{\nu_l^{(1)}}$ carries information about Δ_l. In Fig. 14(d), Recovery using SCA (and Eq. 8 of the main text with $\sqrt{\nu_l^{(1)}}$ instead of $\nu_l^{(1)}$) is 0.97, which remains lower than Recovery using ICOD, which is 0.999 here. Besides, Fig. 15 illustrates that Recovery of $\vec{\Delta}$ by SCA is much better using $\sqrt{\nu_l^{(1)}}$ than $\nu_l^{(1)}$.

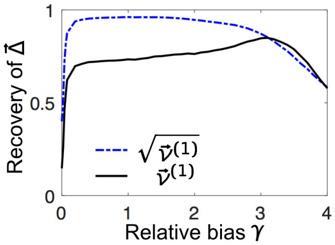

Fig 15. Recovery of $\vec{\Delta}$ from the first SCA eigenvector using $\vec{\nu}^{(1)}$ or $\sqrt{\vec{\nu}^{(1)}}$. The sequence data are the same as used for the blue curves in Fig. 3(a) of the main text. As suggested by Eq. 68, use of the square root of $\vec{\nu}^{(1)}$ significantly improves Recovery.

5.3 Comparison between ICOD and SCA

In the main text, we compared the performance of ICOD and SCA with respect to Recovery of mutational-effect vectors $\vec{\Delta}$ in synthetic data (see Fig. 3 of the main text). We found that ICOD performs well over a broader range of relative biases γ than SCA. The failure of SCA at biases close to zero can be explained by the fact that the conservation weights ϕ_l then vanish (see Eq. 66). A further example of the failure of SCA for non-biased selections is given by the case studied in Fig. 5, where we considered two selections, a biased one associated to $\vec{\Delta}_1$ and a non-biased one associated to $\vec{\Delta}_2$. Fig. 16 shows that SCA recovers $\vec{\Delta}_1$ well, but performs badly for $\vec{\Delta}_2$, while ICOD recovers both of them very well (see Fig. 5).

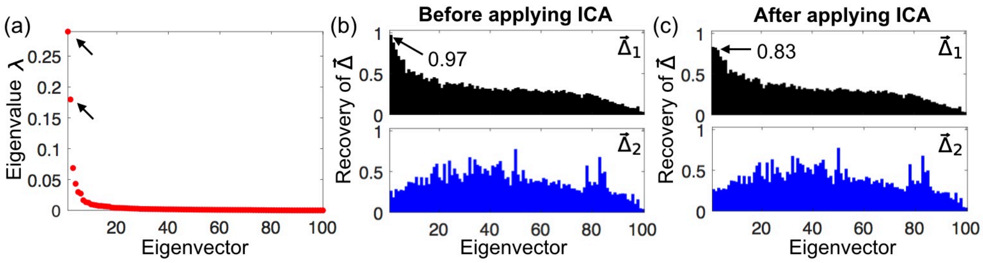

Fig 16. Performance of SCA for the double selection from Fig. 5. (a) Eigenvalues. (b) Before applying ICA, the first eigenvector has high Recovery of $\vec{\Delta}_1$, but no eigenvector has substantial Recovery of $\vec{\Delta}_2$. This difference matches our observation that SCA performs well for selections of intermediate bias, but not for unbiased selections. (c) Applying ICA on the first two eigenvectors does not improve Recovery.

While the comparison of Recovery favors ICOD, SCA was originally used to identify sectors (in our model, sites with important mutational effects under a given selection) rather than to recover complete mutational effect vectors

$\vec{\Delta}$. Hence, in Fig. 17, we compare the ability of ICOD and SCA to predict the n sites with the largest mutational effects. Note that this comparison is independent of whether we use $\vec{\nu}^{(1)}$ or $\sqrt{\vec{\nu}^{(1)}}$ as the predictor in SCA, since the square-root function is increasing and preserves order. Using this criterion, we again find that ICOD performs well over a broad range of relative biases γ, while SCA only works well for sequences selected under moderate biases.

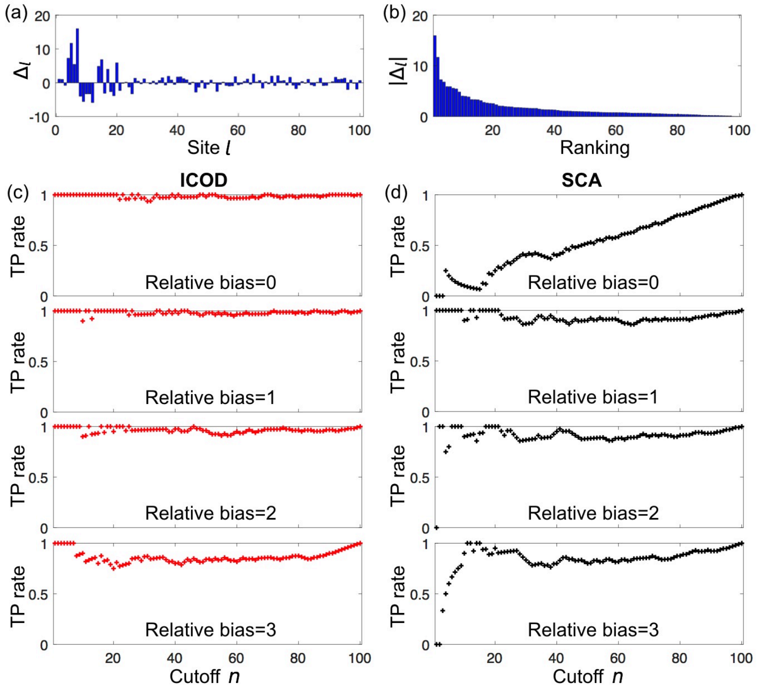

Fig 17. Comparison of sector-site identification by ICOD and SCA (see also Fig. 3 of the main text). We use the synthetic $\vec{\Delta}$ in (a) to selectively weight 5,000 random sequences at four relative bias values $\gamma \equiv (T^* - \langle T \rangle)/\sqrt{\langle (T - \langle T \rangle)^2 \rangle} = 0, 1, 2, 3$ and test the ability of ICOD or SCA to correctly predict the sites with the n largest mutational effects. (b) Magnitudes of mutational effects of $\vec{\Delta}$ by rank. (c-d). True Positive (TP) rates obtained by taking the first eigenvector $\vec{\nu}^{(1)}$ from either ICOD or SCA, generating a ranked list of sites of descending $|\nu_l^{(1)}|$ at each site l, and computing the fraction of the top n sites in this predicted ordering that are also among the top n sites of the actual ordering of mutational effect magnitudes $|\Delta_l|$. The effect of relative bias γ on Recovery is shown in Fig. 3 of the main text. (c) As expected, the prediction of ICOD is very good under all relative biases. (d) On the other hand, SCA does not perform well at the smallest or largest relative biases.

6 Performance of a method based on the generalized Hopfield model

As mentioned in the main text, we also compared ICOD with another PCA-based approach developed in Ref. [4], which employs an inference method specific to the generalized Hopfield model. For L Ising spins ($s_l \in \{-1, 1\}$ for $1 \le l \le L$), the Hamiltonian of the generalized Hopfield model reads (see Eq. 6 in Ref. [4])

$$H(\vec{s}) = -\sum_{l=1}^{L} h_l\, s_l - \frac{1}{2L} \sum_{i=1}^{N} \left(\sum_{l=1}^{L} \xi_{i,l}\, s_l \right)^2 + \frac{1}{2L} \sum_{i=1}^{N'} \left(\sum_{l=1}^{L} \xi'_{i,l}\, s_l \right)^2 , \tag{69}$$

where h_l is the local field at site l, while $\vec{\xi_i} = (\xi_{i,1}, \ldots, \xi_{i,L})$ is an attractive pattern and $\vec{\xi'}_i = (\xi'_{i,1}, \ldots, \xi'_{i,L})$ is a repulsive pattern. Here there are N attractive patterns and N' repulsive ones. In our model, in the single-selection case, the fitness of a sequence $\vec{s}$ in the Ising representation reads (see above, Sec. 3.1.1, Eq. 15)

$$w(\vec{s}) = -\frac{\kappa}{2} \left(\sum_{l=1}^{L} D_l s_l - \alpha \right)^2 = -\frac{\kappa}{2} \left[\left(\sum_{l=1}^{L} D_l s_l \right)^2 - 2\alpha \sum_{l=1}^{L} D_l s_l + \alpha^2 \right] , \tag{70}$$

with $D_l = \Delta_l/2$ and $\alpha = T^* - \sum_l D_l$. Recalling that fitnesses and Hamiltonians have opposite signs, a comparison of Eqs. 69 and 70 shows that $\vec{\Delta}$ plays the part of a repulsive pattern in the two-body coupling terms, with the exact

correspondence given by $\vec{\xi'} = \vec{\Delta}\sqrt{\kappa L}/2$. Note that in our model the local fields are proportional to the components of $\vec{\Delta}$.

Ref. [4] proposed a method to infer attractive and repulsive patterns from data generated using a generalized Hopfield model Eq. 69. Introducing the correlation matrix G, which is related to the covariance matrix C through

$$G_{ll'} = \frac{C_{ll'}}{\tilde{\sigma}_l \tilde{\sigma}_{l'}}, \tag{71}$$

where $\tilde{\sigma}_l^2 = \langle s_l^2 \rangle_* - \langle s_l \rangle_*^2 = 1 - \langle s_l \rangle_*^2$. Ref. [4] found, to lowest order, the following approximation for a single repulsive pattern $\vec{\xi'}$ (see Eq. 9 in Ref. [4]):

$$\xi'_l \approx \sqrt{L\left(\frac{1}{\lambda^{(L)}} - 1\right)} \frac{\nu_l^{(L)}}{\tilde{\sigma}_l}, \tag{72}$$

where $\lambda^{(L)}$ is the smallest (last) eigenvalue of the correlation matrix G and $\nu_l^{(L)}$ is the associated eigenvector. This yields

$$\Delta_l \propto \frac{\nu_l^{(L)}}{\tilde{\sigma}_l}. \tag{73}$$

Inference of $\vec{\Delta}$ based on Eq. 73 is referred to as GHI (for Generalized Hopfield Inference) below.

GHI performs very well for the sequence ensembles from the elastic network model used in Fig. 1 and Fig. 2 of the main text (Fig. 18). Importantly, just as for simple PCA and for ICOD (see main text), the top Recovery is obtained for the (modified) bottom eigenvector of the covariance matrix, consistently with $\vec{\Delta}$ being a repulsive pattern, but the large-eigenvalue modes also contain some information about $\vec{\Delta}$ (Fig. 18).

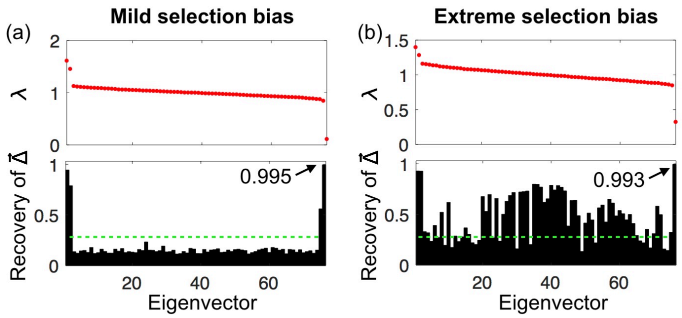

Fig 18. Performance of GHI on sequence ensembles generated with our elastic-network $\vec{\Delta}$. (a) Eigenvalues of G and Recovery under mild selection bias, as in Fig. 1 in the main text. (b) Eigenvalues of G and Recovery under extreme selection bias, as in Fig. 2 in the main text. The green dashed lines in (a,b) indicate the random expectation of Recovery (Eq. 11).

In Fig. 19, we systematically compare all methods discussed in our work to recover $\vec{\Delta}$ from sequence data under various selection biases, using different sector sizes, for selectively weighted ensembles of 50,000 random sequences. We focus on the case of a single selection and compare Recovery of $\vec{\Delta}$ according to:

- ICOD, using the first eigenvector of the modified inverse covariance matrix $\tilde{C}^{-1}$ (see main text, Eq. 10)
- PCA, using the last principal component of the data (last eigenvector of the covariance matrix, see main text)
- SCA, using the first eigenvector of the absolute value of a conservation-weighted covariance matrix, $\tilde{C}_{ll'}^{(\mathrm{SCA})} = |\phi_l C_{ll'} \phi_{l'}|$ (see main text and Ref. [16])
- GHI, using the reweighted last eigenvector of the correlation matrix (see Eqs. 71 and 73).

Overall, ICOD and GHI perform best. For small selection biases, all methods perform accurately, except SCA, which fails when selection bias vanishes, as explained above. When the sector size is small compared to the sequence length L (Fig. 19 (a-d)), GHI performs a little bit better than ICOD for relatively small selection biases (however Recovery remains $\gtrsim 95\%$ with ICOD). Conversely, GHI is significantly outperformed by ICOD for relatively large selection bias, and the performance of PCA and SCA falls off quite rapidly in this regime. The performances of ICOD, PCA, and GHI become similar when the sector size becomes comparable to the sequence length (Fig. 19 (e, f)).

We further find that GHI is more sensitive to the size of the sequence ensemble than ICOD, although it becomes the most accurate for very large dataset sizes (see Fig. 20). The performance of ICOD is quite robust to dataset size. Note that PCA outperforms other methods when the data size becomes very small (Fig. 20, number of sequences = 500).

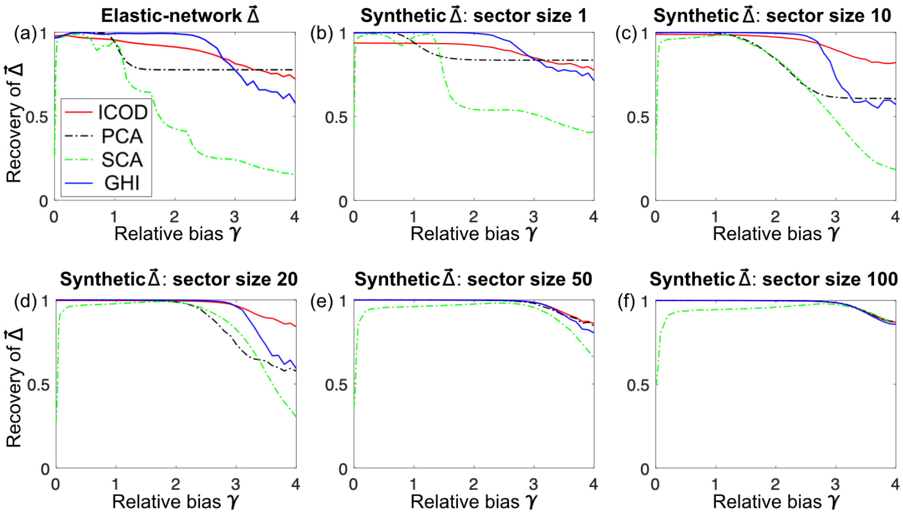

Fig 19. Comparing Recovery of different methods for various $\vec{\Delta}$s. Here, GHI refers to inference based on Eq. 73. Curves are obtained by averaging over 100 realizations, each for an ensemble of 50,000 random sequences. For synthetic $\vec{\Delta}$s, each realization corresponds to a new $\vec{\Delta}$.

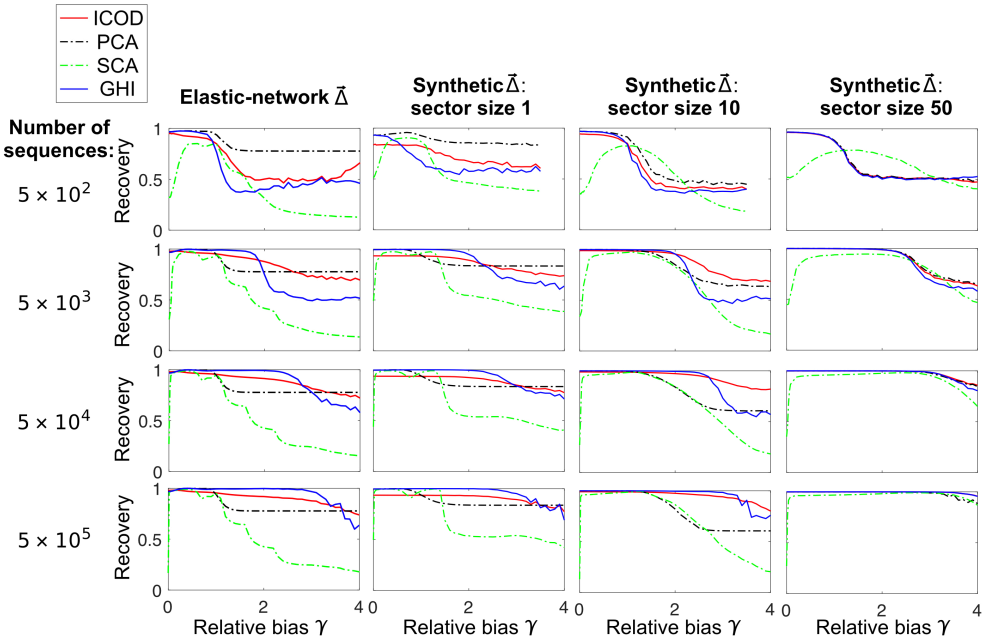

Fig 20. Effect of dataset size on Recovery of $\vec{\Delta}$. Selectively reweighted ensembles of 5×10^2, 5×10^3, 5×10^4, and 5×10^5 random sequences are generated for the elastic-network $\vec{\Delta}$ and synthetic $\vec{\Delta}$s with sector sizes 1, 10, and 50. All results are averaged over 100 realizations, except those using 5×10^5 sequences, where only 5 realizations were used. For synthetic $\vec{\Delta}$s, each realization employs a different $\vec{\Delta}$ with the same sector size. For the case of 500 sequences, some Recoveries were not computed at high biases due to numerical instabilities.

Overall, we find that GHI is very well suited to infer $\vec{\Delta}$ from very large synthetic datasets. However, ICOD is more robust to variation of dataset size and to selection bias, which should be an advantage in the application to real protein data.

7 Application of ICOD to a multiple sequence alignment of PDZ domains

Our general physical model for sectors provides insights into the statistical signatures of sectors in sequence data. In particular, we have found that the primary signature of physical sectors lies in the modes associated with the smallest eigenvalues of the covariance matrix, even though there is often additional signal from these sectors in the large eigenvalue modes, as studied more conventionally, e.g. in SCA. The success of ICOD on synthetic data demonstrates that information about sectors can indeed be extracted from the small eigenvalue modes of the covariance matrix.

How well does ICOD perform on real sequence data? Here, we apply ICOD to an actual alignment of sequences of PDZ domains from the Pfam database (`https://pfam.xfam.org/`) containing 24,934 sequences of length $L = 79$ (corresponding to sites 313-391 in the numbering in Fig. 2 of Ref. [18]). In Ref. [18], sites important for the specific binding of PDZ to peptide ligands were identified experimentally via complete single-site mutagenesis. In particular, 20 sites showing particularly high mutational effects were deemed functionally significant [18]. It was further shown that 15 among the 20 sector amino acids found by SCA (i.e. 75%) were also functionally significant sites.

In order to compute the empirical covariance matrix of the data, we first removed sites with more than 15% gaps (11 sites out of 79). To eliminate the confounding effects of very rare residues at particular sites, we used a pseudocount weight $\Lambda = 0.02$.

Next, we performed both SCA and ICOD using this empirical covariance matrix:

- For SCA, we computed the conservation reweighting factors as in Refs. [11,16], using the background frequency values from Ref. [16]. We compressed the conservation-reweighted covariance matrix using the Frobenius norm, and we focused on the first eigenvector of this reweighted and compressed covariance matrix in order to predict sector sites. Finally, we took the square root of each component of this eigenvector to predict the mutational effect at each site (see above, Section 5.2, and Ref. [11]).
- For ICOD, we inverted the covariance matrix and set its diagonal blocks to zero, thus obtaining the ICOD-modified inverse covariance matrix (see Eq. 49). Next, we computed the Frobenius norm of each 20×20 block associated to each pair of sites (i, j) according to Eq. 52. The magnitude of the l-th component of the first eigenvector $\vec{\nu}^{(1)}$ of this compressed $L \times L$ matrix, denoted by $||\nu_l^{(1)}||$, is the ICOD prediction of the overall mutational effect at site l (see above, Section 4.3, especially Fig. 12). Since mutational effects were experimentally measured with respect to the wild-type residues [18], we used as reference the wild-type sequence of the PDZ domain employed in Fig. 1 of the main text and retained this reference-sequence gauge to perform ICOD, thus allowing direct comparison to experiments.

We then assessed the ability of SCA and ICOD to predict experimentally-measured mutational effects [18]. Specifically, we compared SCA and ICOD predictions to the overall mutational effects corresponding to the Frobenius norm of the experimentally-measured residue-specific mutational effects $\Delta_l(\alpha)$ with $\alpha \in \{1, \dots, 20\}$:

$$||\Delta_l|| = \sqrt{\sum_{\alpha=1}^{q} (\Delta_l(\alpha))^2}, \tag{74}$$

which is the counterpart in the reference-sequence gauge of the "site significance" introduced in the zero-sum gauge in Eq. 55. The ability of SCA and ICOD to identify the sites with the experimentally most important mutational effects is shown in Fig. 5 in the main text. Here, we discuss the impact of parameters on these results. Fig. 21 shows the effect of varying the cutoff for removal of sites with a large proportion of gaps. As illustrated in panel (a), sites with a fraction of gaps larger than a cutoff are discarded. Many of these sites are on the edges of the PDZ domain, and tend to be less conserved. Fig. 21(b) shows that ICOD performance is robust to variations of this cutoff within a reasonable range. We have chosen a cutoff of 15% in the rest of this analysis.

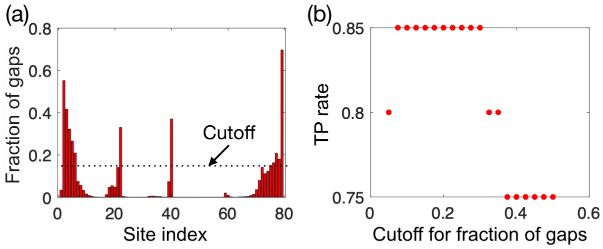

Fig 21. Impact of varying the cutoff for removal of sites with a large fraction of gaps. (a) Fraction of sequences that have a gap at each site. Sites with a fraction of gaps larger than the cutoff shown by the dashed line are discarded in the rest of our analysis. (b) Impact of varying the gap-fraction cutoff on the performance of ICOD. A pseudocount weight of $\Lambda = 0.02$ is used.

Fig. 22 shows the effect of varying the pseudocount weight, both for ICOD and for SCA. Panels (a) and (b) show the TP rate, defined as the fraction of the top 20 predicted sites that are among the 20 sites with the largest experimentally-determined mutational effects. Panels (c) and (d) show the Pearson correlation between ICOD or SCA predictions of mutational effects and the corresponding experimental measurements. Both ICOD and SCA identify experimentally-important sites significantly better than random expectation over the whole range of pseudocounts shown. However, ICOD performs best with small but nonzero pseudocount weights (panels (a) and (c)), while the performance of SCA is more robust to changing the pseudocount weight (panels (b) and (d)).

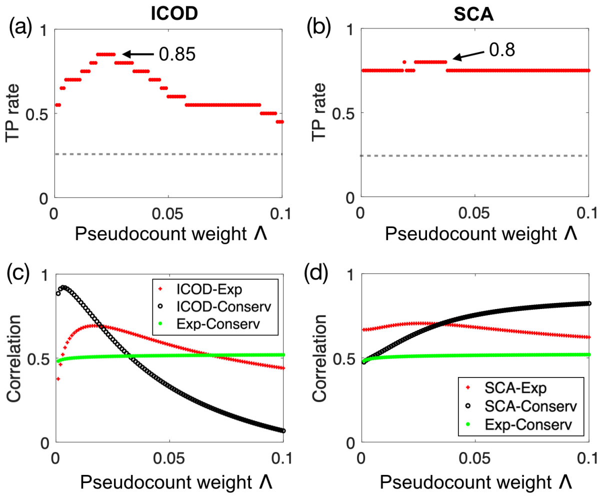

Fig 22. Impact of the pseudocount weight Λ on the performance of ICOD and SCA. (a) Fraction of the 20 top sites predicted by ICOD that are among the 20 sites with the largest experimentally-determined mutational effects ("TP rate") versus pseudocount weight. The TP rate definition is the same as that shown in Fig. 5 of the main text. Gray dashed line: random expectation for the TP rate, namely 20/68=0.29 (68 sites are left after removing those with a gap fraction larger than the cutoff). (b) Counterpart of (a) for SCA. (c) Pearson correlation: between mutational effects predicted by ICOD and those measured experimentally at each site (ICOD-Exp; data from Fig. 5(c) of the main text for $\Lambda = 0.02$); between mutational effects predicted by ICOD and conservation scores ϕ_l (ICOD-Conserv; see Ref. [11] and Eq. 62 in the binary case); and between experimentally measured mutational effects and conservation scores ϕ_l (Exp-Conserv). (d) Counterpart of (b) for SCA. In all panels, a gap-fraction cutoff of 15% is used.

Since residue conservation plays a very important part in the PDZ sector [17], we compared prediction based simply on conservation to those of SCA and ICOD. We employed the conservation scores ϕ_l used in SCA [11], which are a generalization of Eq. 62 to 21 states. Conservation alone identifies 70% of the 20 sites with largest experimentally-determined mutational effects, versus 85% for ICOD (for $\Lambda = 0.02$) and 75% for SCA (see Fig. 5 in the main text). In addition, the Pearson correlation between conservation scores and experimentally-measured mutational effects is significant (see Fig. 23(c)), even though it is smaller than between ICOD or SCA scores and experimentally-measured mutational effects (see Fig. 23(a-b)). In fact, both ICOD and SCA scores are significantly correlated with conservation scores (see Fig. 23 (d-e)). In the case of SCA, this is not surprising given that

conservation scores are explicitly used to weight the covariance matrix. Interestingly, ICOD naturally identifies these conserved sites as being important. This correlation between ICOD and conservation highlights the ability of ICOD to identify functionally important amino acids in a principled way that only relies on covariance.

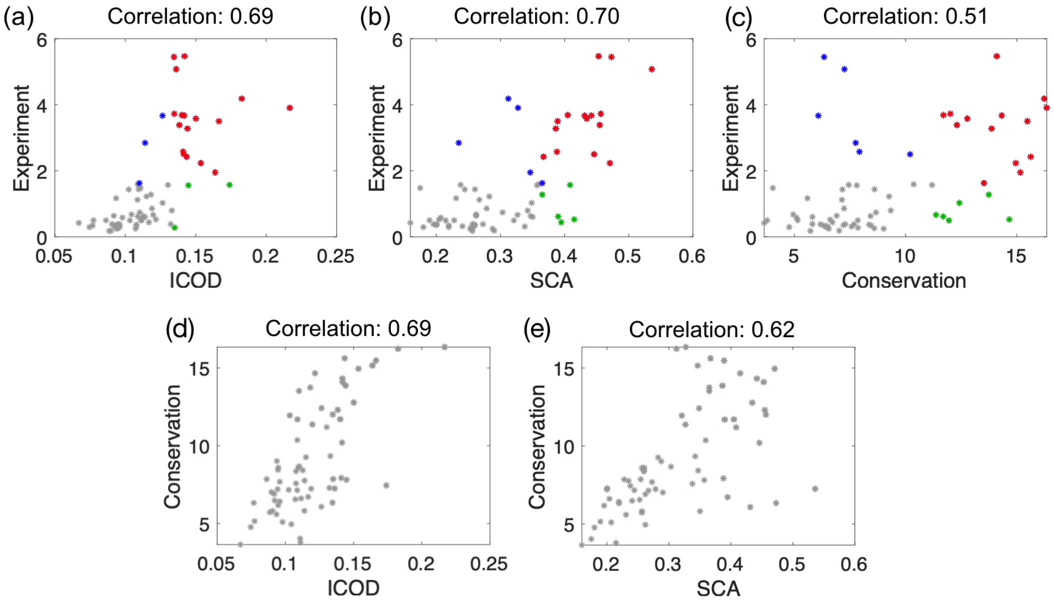

Fig 23. Predicting experimentally-measured mutational effects using ICOD, SCA, or conservation. (a) Experimentally-measured mutational effect versus mutational effect predicted by ICOD for each site of the PDZ sequence. (b) Experimentally-measured mutational effect versus mutational effect predicted by SCA for each site of the PDZ sequence. (c) Experimentally-measured mutational effect versus Conservation score ϕ_l for each site of the PDZ sequence. In panels (a, b, c), to highlight the matches between the top 20 predictions and the top 20 experimentally important sites [18], correct hits are shown in red, false negatives in blue, and false positives in green. (d) Conservation score ϕ_l versus mutational effect predicted by ICOD for each site of the PDZ sequence. (e) Conservation score ϕ_l versus mutational effect predicted by SCA for each site of the PDZ sequence. In all panels, a pseudocount weight $\Lambda = 0.02$ and a gap-fraction cutoff of 15% were used.

References

1. Marks DS, Colwell LJ, Sheridan R, Hopf TA, Pagnani A, Zecchina R, et al. Protein 3D structure computed from evolutionary sequence variation. PLoS ONE. 2011;6(12):e28766.

2. Morcos F, Pagnani A, Lunt B, Bertolino A, Marks DS, Sander C, et al. Direct-coupling analysis of residue coevolution captures native contacts across many protein families. Proc Natl Acad Sci USA. 2011;108(49):E1293–E1301.

3. Plefka T. Convergence condition of the TAP equation for the infinite-ranged Ising spin glass model. J Phys A: Math Gen. 1982;15(6):1971–1978.

4. Cocco S, Monasson R, Sessak V. High-dimensional inference with the generalized Hopfield model: principal component analysis and corrections. Phys Rev E. 2011;83(5 Pt 1):051123.

5. Cocco S, Monasson R, Weigt M. From principal component to direct coupling analysis of coevolution in proteins: Low-eigenvalue modes are needed for structure prediction. PLOS Comput Biol. 2013;9(8):e1003176.

6. Thouless DJ, Anderson PW, Palmer RG. Solution of'solvable model of a spin glass'. Philos Mag. 1977;35(3):593–601.

7. Ekeberg M, Hartonen T, Aurell E. Fast pseudolikelihood maximization for direct-coupling analysis of protein structure from many homologous amino-acid sequences. J Comput Phys. 2014;276:341–356.

8. Ekeberg M, Lövkvist C, Lan Y, Weigt M, Aurell E. Improved contact prediction in proteins: using pseudo-likelihoods to infer Potts models. Phys Rev E. 2013;87(1):012707.

9. Hyvärinen A, Karhunen J, Oja E. Independent Component Analysis. John Wiley and Sons; 2001.

10. Hansen LK, Larsen J, Kolenda T. Blind Detection of Independent Dynamic Components. In: IEEE International Conference on Acoustics, Speech, and Signal Processing 2001. vol. 5; 2001. p. 3197–3200.

11. Rivoire O, Reynolds KA, Ranganathan R. Evolution-Based Functional Decomposition of Proteins. PLoS Comput Biol. 2016;12(6):e1004817.

12. Baldassi C, Zamparo M, Feinauer C, Procaccini A, Zecchina R, Weigt M, et al. Fast and accurate multivariate Gaussian modeling of protein families: predicting residue contacts and protein-interaction partners. PLoS ONE. 2014;9(3):e92721.

13. Weigt M, White RA, Szurmant H, Hoch JA, Hwa T. Identification of direct residue contacts in protein-protein interaction by message passing. Proc Natl Acad Sci USA. 2009;106(1):67–72.

14. Procaccini A, Lunt B, Szurmant H, Hwa T, Weigt M. Dissecting the specificity of protein-protein interaction in bacterial two-component signaling: orphans and crosstalks. PLoS ONE. 2011;6(5):e19729.

15. Bitbol AF, Dwyer RS, Colwell LJ, Wingreen NS. Inferring interaction partners from protein sequences. Proc Natl Acad Sci USA. 2016;113(43):12180–12185.

16. Halabi N, Rivoire O, Leibler S, Ranganathan R. Protein sectors: evolutionary units of three-dimensional structure. Cell. 2009;138(4):774–786.

17. Teşileanu T, Colwell LJ, Leibler S. Protein sectors: statistical coupling analysis versus conservation. PLoS Comput Biol. 2015;11(2):e1004091.

18. McLaughlin Jr RN, Poelwijk FJ, Raman A, Gosal WS, Ranganathan R. The spatial architecture of protein function and adaptation. Nature. 2012;491(7422):138.
